# Colistin-Resistant *Enterobacteriaceae* Isolated From Process Waters and Wastewater From German Poultry and Pig Slaughterhouses

**DOI:** 10.3389/fmicb.2020.575391

**Published:** 2020-10-30

**Authors:** Mykhailo Savin, Gabriele Bierbaum, Khald Blau, Marijo Parcina, Esther Sib, Kornelia Smalla, Ricarda Schmithausen, Céline Heinemann, Jens A. Hammerl, Judith Kreyenschmidt

**Affiliations:** ^1^Institute of Animal Sciences, University of Bonn, Bonn, Germany; ^2^Institute for Hygiene and Public Health, Medical Faculty, University of Bonn, Bonn, Germany; ^3^Institute for Medical Microbiology, Immunology and Parasitology, Medical Faculty, University of Bonn, Bonn, Germany; ^4^Julius Kühn-Institut, Federal Research Centre for Cultivated Plants, Braunschweig, Germany; ^5^Department for Biological Safety, German Federal Institute for Risk Assessment, Berlin, Germany; ^6^Department of Fresh Produce Logistics, Hochschule Geisenheim University, Geisenheim, Germany

**Keywords:** colistin resistance, *mcr* genes, slaughterhouse, wastewater, zoonotic microorganisms, *Escherichia coli*, *Klebsiella pneumoniae*, *Enterobacter cloacae* complex

## Abstract

Due to the high prevalence of colistin-resistant *Enterobacteriaceae* in poultry and pigs, process waters and wastewater from slaughterhouses were considered as a hotspot for isolates carrying plasmid-encoded, mobilizable colistin resistances (*mcr* genes). Thus, questions on the effectiveness of wastewater treatment in in-house and municipal wastewater treatment plants (WWTPs) as well as on the diversity of the prevailing isolates, plasmid types, and their transmissibility arise. Process waters and wastewater accruing in the delivery and unclean areas of two poultry and two pig slaughterhouses were screened for the presence of target colistin-resistant bacteria (i.e., *Escherichia coli*, *Klebsiella* spp., *Enterobacter cloacae* complex). In-house and municipal WWTPs (mWWTPs) including receiving waterbodies were investigated as well. Samples taken in the poultry slaughterhouses yielded the highest occurrence of target colistin-resistant *Enterobacteriaceae* (40.2%, 33/82), followed by mWWTPs (25.0%, 9/36) and pig slaughterhouses (14.9%, 10/67). Recovered isolates exhibited various resistance patterns. The resistance rates using epidemiological cut-off values were higher in comparison to those obtained with clinical breakpoints. Noteworthy, MCR-1-producing *Klebsiella pneumoniae* and *E. coli* were detected in scalding waters and preflooders of mWWTPs. A total of 70.8% (46/65) of *E. coli* and 20.6% (7/34) of *K. pneumoniae* isolates carried *mcr-1* on a variety of transferable plasmids with incompatibility groups IncI1, IncHI2, IncX4, IncF, and IncI2 ranging between 30 and 360 kb. The analyzed isolates carrying *mcr-1* on transferable plasmids (*n* = 53) exhibited a broad diversity, as they were assigned to 25 different *Xba*I profiles. Interestingly, in the majority of colistin-resistant *mcr*-negative *E. coli* and *K. pneumoniae* isolates non-synonymous polymorphisms in *pmrAB* were detected. Our findings demonstrated high occurrence of colistin-resistant *E. coli* and *K. pneumoniae* carrying *mcr-1* on transferrable plasmids in poultry and pig slaughterhouses and indicate their dissemination into surface water.

## Introduction

Since the 1950s, colistin (polymyxin E) has been extensively used in the European animal production (Koyama et al., 1950) to prevent and treat gastrointestinal infections caused by Gram-negative bacteria (e.g., diarrhea in pigs caused by *Escherichia coli* and *Salmonella* spp. as well as colibacillosis in poultry) (EMEA, 2002). Moreover, it was also used in a lower dosage as a feed additive until the ban of antimicrobial growth promoters in the European Union (EU) in 2006 (EMA, 2016).

Despite its nephrotoxicity and neurotoxicity, colistin was re-introduced into human therapy to treat infections caused by multidrug-resistant *Acinetobacter baumannii* and *Pseudomonas aeruginosa* or carbapenemase*-*producing *Enterobacteriaceae* (CPE) (Azzopardi et al., 2013). Due to its high impact, the World Health Organisation (WHO) included colistin into the group of the “highest priority critically important antimicrobials” for human medicine (World Health Organization, 2019). Alongside with other antibiotics of the last resort (e.g., tigecycline, amikacin and the new combinations of ceftazidime-avibactam and ceftozolane-tazobactam), its use is restricted to clinical cases for which no alternative options are available (Nation and Li, 2009). However, in 2016, colistin was also classified as a highly important antimicrobial (VHIA) in the veterinary sector by the World Organisation for Animal Health (OIE, 2018). Data from Germany indicate a reduction of colistin sales between 2011 and 2016 by 45.7% from 127 to 69 tons. However, from 2016 its sales have been slightly increasing and reached 74 tons in 2018 making up 10.2% of the total amount of antimicrobials sold for the veterinary use in Germany (BVL, 2019).

In Gram-negative bacteria, colistin interacts with lipopolysaccharide (LPS) and phospholipids in the outer cell membrane. Due to the competitive displacement of divalent cations Ca^2+^ and Mg^2+^ from the phosphate groups of membrane lipids (Falagas and Kasiakou, 2005), both cell membranes are disrupted leading to the leakage of intracellular contents and subsequent bacterial death.

Before 2015, colistin resistance in *Enterobacteriaceae* was assumed to be caused due to chromosomal mutations in genes (esp. *pmrA/B* and *phoP/Q* and *mgrB*) encoding regulatory proteins that influence transcription of enzymes that modify the lipopolysaccharide (Moskowitz et al., 2012; Quesada et al., 2015). But the description of the first plasmid-encoded, mobilizable colistin resistance gene (*mcr-1*) in *E. coli* from livestock in China and retail meat as well as in Chinese clinical *Klebsiella pneumoniae* isolates (Liu et al., 2016; Sun et al., 2018b) raised serious public health concern on the emergence of colistin-resistant bacteria.

Further studies on the genetic basis of colistin-resistant bacteria resulted in the discovery of nine additional *mcr* genes (*mcr-2* to *mcr-10*). However, *mcr-1* is the most prevalent worldwide (Elbediwi et al., 2019). *mcr*-occurrence is often associated with a variety of plasmids, including IncX4, IncF, IncHI1, IncHI2, IncI2, IncY, and broad host range (BHR) plasmids IncP (Zurfuh et al., 2016; Hadjadj et al., 2017; Poirel et al., 2017; Sun et al., 2018a). Furthermore, *mcr-1* is often bracketed by IS*Apl1* insertion sequence enabling their broad dissemination by transposition (Snesrud et al., 2016; Li et al., 2017).

Due to a high number of colonized animals, slaughterhouses might represent a significant source of introduction of *mcr* genes into the food chain, e.g., through possible contamination of carcasses and products (Irrgang et al., 2016; Inderbinen, 2017). Furthermore, slaughterhouse workers with occupational exposure to colonized animals and contaminated process water as well as employees of the wastewater treatment plants (WWTPs) might be exposed to an increased risk of colonization (Dohmen et al., 2017). Moreover, due to insufficient wastewater treatment by in-house and municipal WWTPs (mWWTPs), livestock wastewater might be an important route for dissemination of *mcr-1*-carrying bacteria into the environment (Hembach et al., 2017).

On the basis of the high prevalence of colistin-resistant *Enterobacteriaceae* in livestock feces, these bacteria might accumulate in process waters and wastewater from slaughterhouses. These waters might represent potential reservoirs that can contribute to a broad spread of the resistance to other environmental ecosystems including surface waters. So far, no data on the occurrence and characteristics of colistin-resistant *Enterobacteriaceae* in process waters and wastewater from German poultry and pig slaughterhouses have been reported. Furthermore, information on the impact of slaughterhouse wastewaters for the dissemination of this resistance is scarce and needs to be determined. Thus, this study aimed to evaluate their occurrence in the delivery and unclean areas of German poultry and pig slaughterhouses as well as in their in-house WWTPs. Moreover, their further spread into surface waters via municipal WWTPs was also investigated.

This hypothesis was tested using selective culture-dependent methods, followed by phenotypic and molecular characterization of the recovered isolates.

## Materials and Methods

### Sampling and Sample Preparation

Sampling and sample preparation of process waters and wastewater taken in poultry and pig slaughterhouses, their in-house WWTPs as well as mWWTPs and on-site preflooders have been previously described (Savin et al., 2020a,b).

A total of 185 water samples were included in the study. Briefly, 82 samples of process waters and wastewater accruing in the delivery and unclean areas during slaughtering and cleaning operations were collected from two poultry slaughterhouses. Samples were taken at seven sampling sites: transport trucks (*n* = 5), transport crates (*n* = 10), stunning facilities (*n* = 10), scalders (*n* = 10), eviscerators (*n* = 10), production facilities (*n* = 5), influent (*n* = 16), and effluent (*n* = 16) of the in-house WWTPs. From each individual sample, one liter was collected using sterile Nalgene^®^ Wide Mouth Environmental Sample Bottles (Thermo Fisher Scientific, Waltham, MA, United States). For more details please see Savin et al. (2020b).

Further 67 samples of process water and wastewater were collected from the delivery [animal transporters (*n* = 10), holding pens (*n* = 7)] and unclean areas [scalding and dehairing water (*n* = 10), aggregate wastewater from production facilities (*n* = 10)] as well as the in-house WWTPs (in- and effluent, each *n* = 15) of two pig slaughterhouses during slaughtering and cleaning operations. Additionally, 18 samples were collected from the influents (*n* = 9) and effluents (*n* = 9) of the mWWTPs receiving the wastewater from the investigated pig slaughterhouses for the final treatment. Their on-site preflooders upstream (*n* = 9) and downstream the discharge points (*n* = 9) were sampled as well. At each site, one liter was collected in sterile polyethylene Nalgene Wide Mouth Environmental Sample Bottles (Thermo Fisher Scientific, Waltham, MA, United States). For more details please see Savin et al. (2020a).

### Cultivation, Identification and Susceptibility Testing of Target Polymyxin-Resistant Lactose-Fermenting *Enterobacteriaceae*

Water samples were screened for polymyxin-resistant lactose-fermenting *Enterobacteriaceae* (*E. coli*, *Klebsiella* spp., and *Enterobacter cloacae* complex) using SuperPolymyxin medium (Nordmann et al., 2016). For cultivation, aliquots of 100 μl and 1 ml of the original samples were applied onto SuperPolymyxin plates and incubated under aerobic conditions at 37°C for 18–24 h. When possible, up to three colonies of lactose fermenters were picked based on their morphology and sub-cultured on Columbia Agar with 5% sheep blood (MAST Diagnostica, Reinfeld, Germany) at 37°C for 18–24 h.

Identification of the isolates species was conducted by MALDI-TOF MS as previously described (Savin et al., 2020b).

The antimicrobial susceptibility testing of the isolates and transconjugants was performed by applying two different antibiotic susceptibility testing panels as well as epidemiological and clinical breakpoints. The first scheme (A) was based on broth microdilution according to CLSI guidelines (M07-A9) following application of epidemiological cut-off values of European Committee on Antimicrobial Susceptibility Testing (EUCAST) as recommended for isolates from livestock and food. The second one (B) was applied in order to assess the clinical relevance of recovered colistin-resistant isolates in human medicine. For this purpose, they were tested against clinically important antimicrobials for humans by microdilution method as previously described (Savin et al., 2020b). MICRONAUT MIC-Strips Colistin (MERLIN Diagnostika GmbH, Bornheim-Hersel, Germany) were used to test the colistin concentrations of up to 64 mg/L.

Also, isolates of *E. coli*, *K. pneumoniae*, and *E. cloacae* complex that were cultivated from the same samples on CHROMagar^TM^ ESBL plates (MAST Diagnostica, Reinfeld, Germany) as described previously by Savin et al. (2020b) and showed resistance to colistin, were included in this study.

### Molecular Typing of Resistant Bacterial Isolates

Cell lysates prepared by boiling of bacterial suspensions (Aldous et al., 2005) were used as template for PCR. Determination of phylogenetic groups (A, B1, B2, C, D, E, F, clade I–V) of *E. coli* was conducted according to a previously published method (Clermont et al., 2013).

### PCR Screening for *mcr-1* to *mcr-9* Genes and Sanger-Sequencing of the Amplicons

Isolates were screened for *mcr-1* to *mcr-5* as well as *mcr-6* to *mcr-9* genes using the multiplex PCR protocols as described by Rebelo et al. (2018) and Borowiak et al. (2020), respectively. As positive controls the isolates *E. coli* R2749 (*mcr-1*), *E. coli* KP37 (*mcr-2*), *Salmonella* Typhimurium SSI_AA940 (*mcr-3*), *S.* Typhimurium R3445 (*mcr-4*), *E. coli* 10E01066 (*mcr-5*), and *S.* Infantis 15-SA01028 (*mcr-9*) were used. The artificially synthesized positive controls for *mcr-6*, *mcr-7*, and *mcr-8* were kindly provided by the Department for Biological Safety of German Federal Institute for Risk Assessment (BfR) (Berlin, Germany) (Borowiak et al., 2020). PCR products were separated by electrophoresis on a 1.0% agarose-TBE gel and stained with midori green (Labomedic Medizin- und Labortechnik GmbH, Bonn, Germany). Sequence-based typing of *mcr-1* (Zhang et al., 2018) amplicons was performed at Microsynth Seqlab (Göttingen, Germany).

### *Xba*I PFGE-Profiling of *mcr-1*-Positive *E. coli* and *K. pneumoniae* Isolates and *mcr-1* Localization

The phylogenetic relationship of the *mcr-1*-carrying *E. coli* and *K. pneumoniae* was assessed by *Xba*I macrorestriction via pulsed-field gel electrophoresis (PFGE) according to the PulseNet protocol (CDC, 2020). Plasmidal localization of the *mcr* genes was determined by S1-PFGE followed by Southern blotting and DNA-DNA hybridization against a digoxigenin-labeled PCR amplicon as previously described (Hammerl et al., 2018). The size of *mcr*-carrying plasmids was predicted on the basis of the S1-PFGE pictures with Bionumerics (Applied Math, Sint Marten-Latem, Netherlands; version 7.5) using *Salmonella* Braenderup (H9812) as size marker.

### Conjugation Assays and Plasmid Analyses

*In vitro* conjugation experiments were conducted in liquid medium using the plasmid-free rifampicin-resistant *E. coli* recipient strain CV601 GFP at a donor:recipient ratio of 1:1 as previously described (Blau et al., 2018). Transconjugants were selected after incubation at 37°C for 24–48 h under selective conditions on lysogeny broth (LB) agar (Sigma-Aldrich, St. Louis, MO, United States) containing colistin sulfate (1 μg/ml) and rifampicin (200 μg/ml) (w/v). Isolates that did not yield transconjugants were further subjected to filter mating assays with the rifampicin-resistant, lactose-negative *E. coli* recipient strain W3110 at a donor:recipient ratio of 10:1 (Kieffer et al., 2017). The selection of transconjugants was done on MacConkey agar (Sigma-Aldrich, St. Louis, MO, United States) containing colistin sulfate (1 μg/ml) and rifampicin (200 μg/ml) after incubation at 37°C for 24–48 h under selective conditions. Potential transconjugants were subjected to PCR to confirm the presence of the *mcr* genes. Those transconjugants obtained with *E. coli* CV601 as recipient were additionally examined for GFP fluorescence using fluorescence microscope Axio Scope.A1 (Carl Zeiss Microscopy GmbH, Jena, Germany).

### Transformation Assays

*mcr-1*-positive isolates that did not generate any transconjugants were further submitted to transformation experiments using NEB^®^ 10-beta electrocompetent *E. coli* cells (New England Biolabs, Ipswich, MA, United States) and MicroPulser Electroporator (Bio-Rad, Hercules, CA, United States) according to manufacturer’s protocols. Plasmid DNA was extracted from overnight cultures of *mcr-1*-positive isolates using GeneJET Plasmid Miniprep Kit (Thermo Fisher Scientific, Waltham, MA, United States) according to manufacturer’s protocol. The transformants were selected on LB agar (Sigma-Aldrich, St. Louis, MO, United States) containing colistin sulfate (1 μg/ml).

The transconjugants and transformants were cryopreserved at −20°C using cryotubes (Mast Diagnostics, Reinfeld, Germany) until further analysis.

### Plasmid Replicon Typing

Plasmid DNA was extracted from overnight cultures of *E. coli* CV601 and W3110 transconjugants using GeneJET Plasmid Miniprep Kit (Thermo Fisher Scientific, Waltham, MA, United States) according to manufacturer’s protocol. The presence of IncF and IncI plasmids was tested by RT-PCR 5′-nuclease assays (TaqMan RT-PCR) as previously described (Blau et al., 2018). Plasmids from transconjugants that could not be detected by RT-PCR were further investigated by PCR-Based Replicon Typing (PBRT). Therefore, PCR amplification on plasmid DNA was performed using primers for the 30 different replicons (HI1, HI2, I1, I2, X1, X2, X3, X4, L, M, N, FIA, FIB, FIC, FII, FIIS, FIIK, FIB KN, FIB KQ, W, Y, P1, A/C, T, K, U, R, B/O, HIB-M, and FIB-M), which are representative for the major plasmid incompatibility groups among *Enterobacteriaceae* (Carattoli et al., 2005; Villa et al., 2010).

### Amplification and Sequencing of *pmrA* and *prmB* Genes in *mcr*-Negative *E. coli* and *K. pneumoniae* Isolates

The coding sequences of the *pmrA* and *pmrB* genes in *E. coli* and *K. pneumoniae* were amplified as previously described by Jayol et al. (2014), Quesada et al. (2015), and Haeili et al. (2017). PCR amplicons were purified using the innuPREP DOUBLEpure Kit (Analytik Jena AG, Jena, Germany) and sequenced at Microsynth Seqlab (Göttingen, Germany). Genomic DNA from five randomly selected *mcr-1*-negative colistin-susceptible *E. coli* and *K. pneumoniae* isolates (colistin MIC < 2 mg/L) originating from the same samples were used as control. Sequence analysis was conducted with Chromas lite v.2.6.5 (Technelysium Pty. Ltd.) and BioEdit v.7.2.5 (Hall, 1999).

## Results

### Detection of *Enterobacteriaceae* in Samples From Poultry and Pig Slaughterhouses as Well as From mWWTPs

Due to the growth of accompanying bacterial microbiota that belongs to intrinsically colistin-resistant genera (e.g., *Proteus, Providencia, Morganella*) and colistin-susceptible isolates on the selective agar plates as well as absence of sample replicates, it was not possible to perform accurate quantification of target bacteria. This could be considered as a limitation of this study.

**FIGURE 1:**
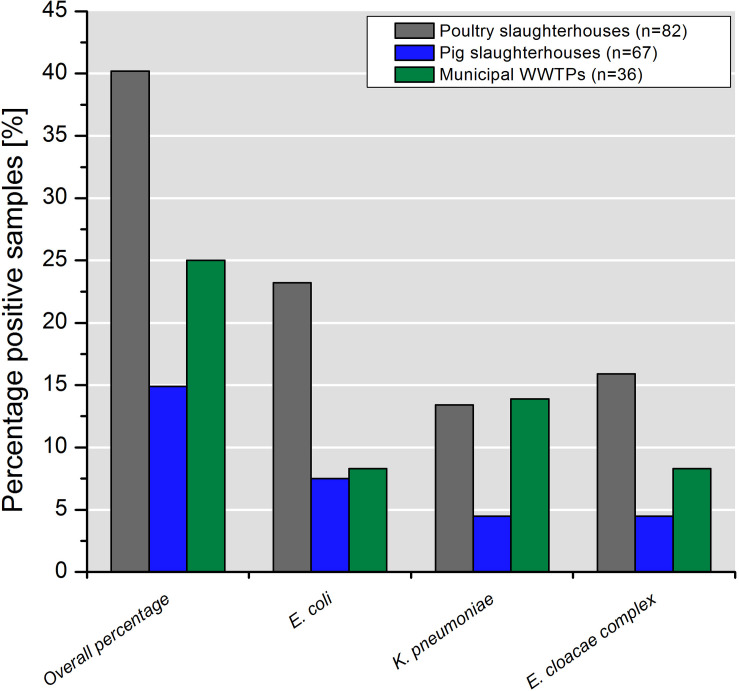
Percentage of samples containing colistin-resistant target bacteria taken in poultry and pig slaughterhouses as well as in the municipal WWTPs.

Water samples collected in poultry slaughterhouses yielded the highest percentage of colistin-resistant *Enterobacteriaceae* (40.2%; 33/82) followed by mWWTPs (25.0%, 9/36) and pig slaughterhouses (14.9%, 10/67). Detailed information on species distribution is shown in Figure 1.

**FIGURE 2:**
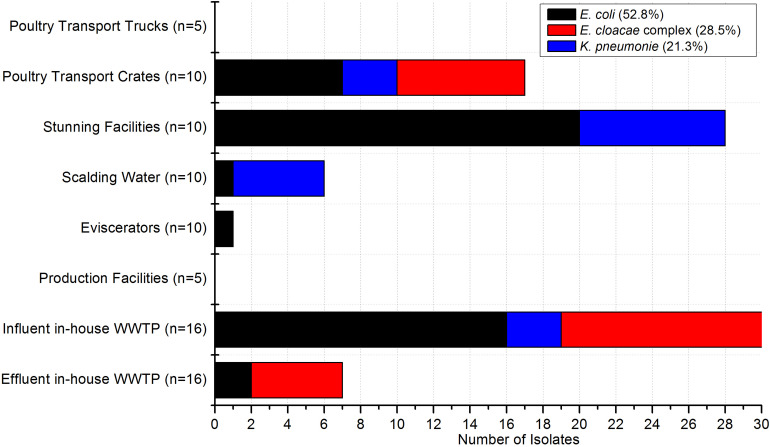
Occurrence of target bacteria tested as colistin-resistant across the sampling points in poultry slaughterhouses (*n* = 82). Number of samples taken at each sampling point is stated.

**FIGURE 3:**
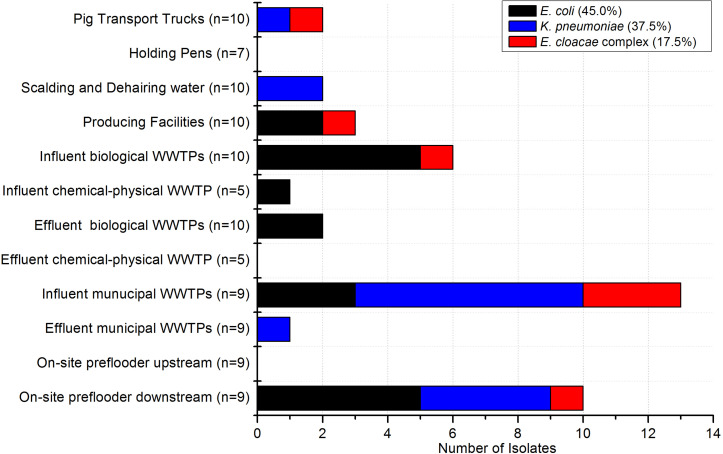
Occurrence of target bacteria tested as colistin-resistant across the sampling points in pig slaughterhouses (*n* = 67) and in the municipal WWTPs receiving wastewater from the investigated pig slaughterhouses (*n* = 36). Number of samples taken at each sampling point is stated.

In the poultry and pig slaughterhouses the target bacteria were recovered at almost all sampling points as shown in Figures 2, 3, respectively. Interestingly, only one out of nine samples taken in the effluent of the mWWTPs was positive for target colistin-resistant bacteria. Moreover, no colistin-resistant target bacteria were detected in the on-site preflooders upstream the discharge point (Figure 3).

Overall, 129 isolates were recovered from 185 samples. Of the isolates, 50.4% were determined as *E. coli*, 26.3% as *K. pneumoniae* and 23.3% as isolates of the *E. cloacae* complex. The most frequently isolated species in poultry and pig slaughterhouses was *E. coli*, whereas in mWWTPs *K. pneumoniae* was more abundant.

### Resistance Patterns [Scheme A (EUCAST) and Scheme B (KRINKO)] and MIC of Colistin per Species

**FIGURE 4:**
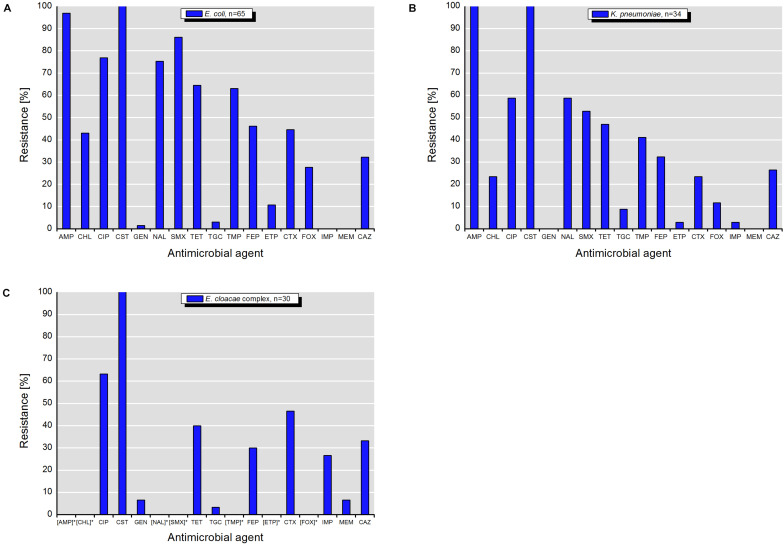
Resistance to antimicrobial agents detected among target colistin-resistant isolates of **(A)** *E. coli*, **(B)** *K. pneumonia*, and **(C)** *E. cloacae* complex with MICs interpreted according to the epidemiological cut-off values (ECOFFs) of EUCAST (scheme A). MICs (mg/L) of antimicrobials with undefined epidemiological cut-offs for *E. cloacae* complex isolates are shown in Table 1. AMP, ampicillin; CHL, chloramphenicol; CIP, ciprofloxacin; CST, colistin; GEN, gentamicin; NAL, nalidixic acid; SMX, sulfamethoxazole; TET, tetracycline; TGC, tigecycline; TMP, trimethoprim; FEP, cefepime; ETP, ertapenem; CTX, cefotaxime; FOX, cefoxitin; IMI, imipenem; MEM, meropenem; CAZ, ceftazidime. []* – antimicrobials with undefined ECOFFs.

**FIGURE 5:**
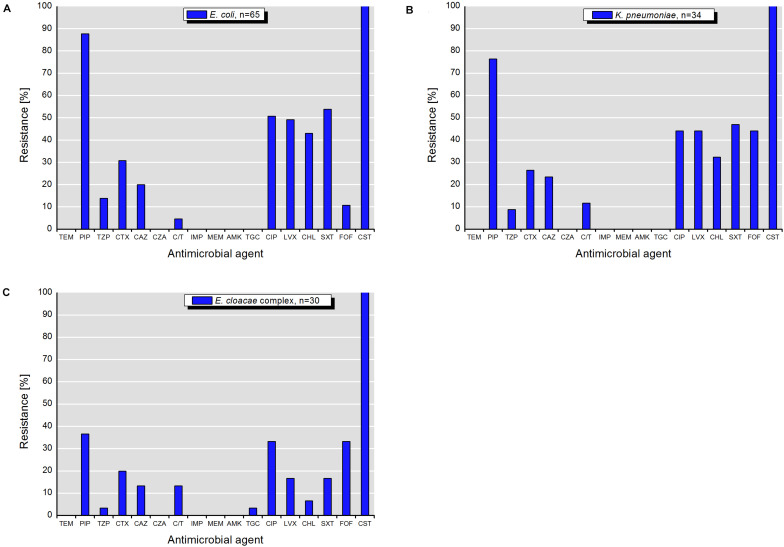
Resistance to antimicrobial agents detected among target colistin-resistant isolates of **(A)** *E. coli*, **(B)** *K. pneumonia*, and **(C)** *E. cloacae* complex with MICs interpreted according to the clinical breakpoints of EUCAST (scheme B). TEM, temocillin; PIP, piperacillin; TZP, piperacillin-tazobactam; CTX, cefotaxime; CAZ, ceftazidime; CZA, ceftazidime-avibactam; C/T, ceftolozane-tazobactam; IMP, imipenem; MEM, meropenem; AMK, amikacin; TGC, tigecycline; CIP, ciprofloxacin; LVX, levofloxacin; CHL, chloramphenicol; SXT, sulfamethoxazole-trimethoprim; FOF, fosfomycin; CST, colistin.

Isolates of *E. coli*, *K. pneumoniae*, and *E. cloacae* complex exhibited various resistance patterns. The resistance rates using epidemiological cut-off values (Figures 4A–C) were higher and different in comparison to those obtained with clinical breakpoints (Figures 5A–C).

According to the scheme A, the recovered isolates were either susceptible or expressed low resistance rates to gentamicin, tigecycline and with exception of *E. cloacae* complex to carbapenems (IMP and MEM). The resistance rates to 3rd generation cephalosporins (CTX and CAZ) varied between isolated species and were in the range of 23.5% for *K. pneumoniae* and 46.7% for *E. cloacae* complex. The highest level of multiple drug resistance (MDR, combined resistance to CST, CIP, and TET) shown isolates of *E. coli* (49.2%), followed by *K. pneumoniae* (35.3%), and *E. cloacae* complex (33.3%). MICs of antimicrobials with undefined epidemiological cut-offs for *E. cloacae* complex (AMP, CHL, NAL, SMX, TMP, ETP, and FOX) are shown in Table 1. MIC values of colistin for recovered *E. coli* and *K. pneumoniae* isolates are shown in Tables 2–4. Among isolates of *E. cloacae* complex, MIC values of colistin varied between 16 and >64 mg/L.

**TABLE 1:** MICs (mg/L) of antimicrobials with undefined epidemiological cut-offs for *E. cloacae* complex isolates tested negative for *mcr-1* to *mcr-9*.

Isolate	Species	Origin	AMP	CHL	NAL	SMX	TMP	ETP	FOX
**Poultry slaughterhouses**									
C-04/10-01	*Enterobacter asburiae*	Effluent in-house WWTP	>64	≤8	>128	≤8	≤0.25	0.03	>64
C-04/02-01	*E. asburiae*	Transport crates	16	16	8	>1024	2	0.03	>64
C-04/02-03	*E. asburiae*	Transport crates	32	≤8	≤4	≤8	0.5	0.03	>64
C-04/02-16	*E. asburiae*	Transport crates	8	16	≤4	256	1	0.03	>64
C-04/02-22	*E. asburiae*	Transport crates	8	16	16	≤8	0.5	0.03	>64
C-04/05-23	*E. asburiae*	Influent in-house WWTP	16	≤8	>128	≤8	≤0.25	0.03	>64
C-04/05-24	*E. asburiae*	Influent in-house WWTP	16	≤8	64	≤8	0.5	0.03	>64
C-04/05-28	*E. asburiae*	Influent in-house WWTP	16	16	16	512	1	0.03	>64
C-04/05-31	*E. asburiae*	Influent in-house WWTP	8	≤8	>128	≤8	≤0.25	≤0.015	>64
C-04/05-33	*E. asburiae*	Influent in-house WWTP	4	≤8	>128	≤8	≤0.25	≤0.015	>64
C-04/05-35	*E. asburiae*	Influent in-house WWTP	8	≤8	>128	≤8	0.5	≤0.015	>64
C-04/05-41	*E. asburiae*	Influent in-house WWTP	8	≤8	>128	≤8	≤0.25	≤0.015	>64
C-04/05-42	*E. asburiae*	Influent in-house WWTP	16	≤8	16	≤8	≤0.25	≤0.015	>64
C-04/05-43	*E. asburiae*	Influent in-house WWTP	8	≤8	>128	≤8	≤0.25	0.06	>64
C-04/05-44	*E. asburiae*	Influent in-house WWTP	16	≤8	16	≤8	≤0.25	≤0.015	>64
C-04/06-04	*E. asburiae*	Effluent in-house WWTP	32	≤8	>128	≤8	≤0.25	0.03	>64
C-04/06-07	*E. asburiae*	Effluent in-house WWTP	32	≤8	≤4	≤8	≤0.25	0.12	>64
C-04/05-29	*Enterobacter hormaechei*	Influent in-house WWTP	>64	≤8	>128	≤8	≤0.25	0.03	>64
C-04/06-05	*E. hormaechei*	Effluent in-house WWTP	>64	≤8	>128	≤8	≤0.25	0.03	>64
C-04/06-01	*Enterobacter kobei*	Effluent in-house WWTP	32	16	≤4	≤8	≤0.25	0.03	>64
01/02-22	*E. asburiae*	Transport crates	>64	≤8	≤4	≤8	≤0.25	0.03	>64
04/02-02	*E. asburiae*	Transport crates	>64	≤8	>128	≤8	1	0.5	>64
04/02-04	*E. asburiae*	Transport crates	>64	16	16	32	1	1	>64

**Pig slaughterhouses and mWWTPs**									

C-03/02-01	*E. asburiae*	Influent biological WWTP	16	≤8	≤4	≤8	≤0.25	≤0.015	>64
C-05/10-24	*E. asburiae*	Influent municipal WWTP	8	≤8	≤4	≤8	≤0.25	0.03	>64
C-05/10-25	*E. asburiae*	Influent municipal WWTP	64	≤8	8	≤8	≤0.25	0.03	>64
C-03/09-03	*E. cloacae*	Producing facilities	16	≤8	≤4	≤8	≤0.25	0.03	>64
05/03-11	*Enterobacter aerogenes*	Pig Transport Trucks	>64	16	≤4	>1024	>32	0.06	>64
03/10-33	*E. asburiae*	Influent municipal WWTP	>64	≤8	>128	>1024	>32	2	>64
03/12-25	*E. asburiae*	On-site preflooder downstream	>64	≤8	>128	>1024	>32	0.12	>64

*AMP, ampicillin; CHL, chloramphenicol; NAL, nalidixic acid; SMX, sulfamethoxazole; TMP, trimethoprim; ETP, ertapenem; FOX, cefoxitin.*

**TABLE 2:** Characteristics of MCR-1–producing *E. coli* and *K. pneumoniae* isolates and their transconjugants.

			Isolates			Transconjugants			
			
Isolate	Species	Origin	Colistin MIC, mg/L	Resistance phenotype (epidemiological cut-off values of EUCAST)^a^	Resistance phenotype (clinical breakpoints of EUCAST)^b^	Incompatibility group (kb) of *mcr-1* plasmids	Colistin MIC of transconjugants mg/L	Co-transferred resistance (epidemiological cut-off values of EUCAST)^a^	Co-transferred resistance (clinical breakpoints of EUCAST)^b^
**Poultry slaughterhouses**									
01/05-11	*E. coli*	Influent in-house WWTP	8	AMP, CHL, CIP, CST, CTX, NAL, SMX, CAZ, TMP, FEP	PIP, CTX, CHL, SXT, CST	IncF (30)	8		
01/05-12	*E. coli*	Influent in-house WWTP	8	AMP, CHL, CIP, CST, CTX, NAL, SMX, CAZ, TMP, FEP	PIP, CTX, CHL, SXT, CST	IncF (30)	8		
01/07-07	*E. coli*	Stunning facilities	8	AMP, CHL, CIP, CST, CTX, NAL, SMX, CAZ, TMP, FEP	PIP, CTX, CHL, SXT, CST	IncX4 (30)	4	CIP, NAL	
01/07-09	*E. coli*	Stunning facilities	8	AMP, CHL, CIP, CST, CTX, NAL, SMX, CAZ, TMP, FEP	PIP, CTX, CHL, SXT, CST	IncI1 (30)	8	CIP, NAL	
01/07-11	*E. coli*	Stunning facilities	8	AMP, CHL, CIP, CST, CTX, NAL, SMX, CAZ, TMP, FEP	PIP, CTX, CAZ, CHL, SXT, CST	IncX4 (30)	4	CIP, NAL	
01/07-12	*E. coli*	Stunning facilities	8	AMP, CHL, CIP, CST, CTX, FOX, NAL, SMX, CAZ, TMP, FEP	PIP, CTX, CHL, SXT, CST	IncX4 (30)	4	CIP, NAL	
01/08-08	*E. coli*	Eviscerators	8	AMP, CHL, CIP, CST, CTX, NAL, SMX, CAZ, TMP, FEP	PIP, CTX, CAZ, CHL, SXT, FOF, CST	IncHI2 (30)	4	CIP, NAL	
04/05-12	*E. coli*	Influent in-house WWTP	4	AMP, CHL, CIP, CST, CTX, FOX, NAL, SMX, CAZ, TET, FEP	PIP, CTX, CAZ, C/T, CIP, LVX, CHL, CST	IncHI2 (245)	4	AMP, CIP, CTX, CAZ	
C-01/05-03	*E. coli*	Influent in-house WWTP	4	AMP, CIP, CST, NAL, SMX, TET, TMP	PIP, CIP, SXT, CST	IncI1 (30)	4	AMP, SMX, TMP	SXT
C-01/05-04	*E. coli*	Influent in-house WWTP	4	AMP, CIP, CST, NAL, SMX, TET, TMP	PIP, CIP, LVX, SXT, CST	IncI2 (n.d.*)	4	CIP, NAL	
C-01/07-02	*E. coli*	Stunning facilities	4	AMP, CIP, CST, NAL, SMX, TET, TMP	PIP, CIP, LVX, SXT, CST	IncX4 (30)	4	CIP, NAL	PIP, CIP, LVX, SXT
C-01/07-04	*E. coli*	Stunning facilities	4	AMP, CIP, CST, NAL, SMX, TET, TMP	PIP, CIP, LVX, SXT, CST	IncX4 (30)	4	CIP, NAL	
C-01/07-06	*E. coli*	Stunning facilities	4	AMP, CIP, CST, NAL, SMX, TET, TMP	PIP, SXT, CST	IncX4 (30)	4	CIP, NAL	
C-04/02-02	*E. coli*	Transport crates	4	AMP, CIP, CST, NAL, SMX, TET	PIP, CIP, LVX, CST	IncX4 (30)	4	CIP, NAL	
C-04/05-10	*E. coli*	Influent in-house WWTP	4	AMP, CST, SMX, TET, TMP	PIP, SXT, CST	IncI1 (360)	4	AMP, SMX, TET, TMP	PIP, SXT
C-04/05-14	*E. coli*	Influent in-house WWTP	4	AMP, CST, SMX, TET, TMP	FOF, CST	IncI1 (360)	4	AMP, SMX, TET, TMP	
C-04/06-02	*E. coli*	Effluent in-house WWTP	8	AMP, CST, SMX, TMP	PIP, CIP, LVX, CST	IncHI2 (30)	8	AMP, SMX, TMP	PIP
C-04/07-04	*E. coli*	Stunning facilities	4	AMP, CIP, CST, SMX, NAL, TET, TMP	PIP, CIP, SXT, CST	IncF (215)	4	AMP, CIP, NAL, SMX, TMP	SXT

**Poultry slaughterhouses**									

C-04/07-07	*E. coli*	Stunning facilities	4	AMP, CIP, CST, SMX, NAL, TET, TMP	PIP, SXT, CST	IncHI2 (215)	4	AMP, CIP, NAL, TET	PIP, SXT
04/07-04	*K. pneumoniae*	Stunning facilities	8	AMP, CIP, CST, CTX, NAL, SMX, CAZ, TET, TMP, FEP, FOX	PIP, CTX, CIP, LVX, CHL, SXT, FOF, CST	IncI1 (30)	4	CIP, NAL	
04/07-12	*K. pneumoniae*	Stunning facilities	8	AMP, CHL, CIP, CST, CTX, FOX, NAL, SMX, CAZ, TET, FEP	PIP, TZP, CTX, CAZ, CIP, LVX, CHL, SXT, FOF, CST	IncX4 (85)	8	CIP, CTX, NAL, CAZ, FOX	
04/07-14	*K. pneumoniae*	Stunning facilities	16^*c*^	AMP, CIP, CST, CTX, NAL, SMX, CAZ, TMP, FEP	PIP, CTX, CAZ, C/T, CIP, LVX, SXT, FOF, CST	IncX4 (30)	4	CIP, NAL	
C-04/02-17	*K. pneumoniae*	Transport crates	>64^*c*^	AMP, CST	PIP, SMX, CST	IncI1 (30)	8		
C-04/03-01	*K. pneumoniae*	Scalding water	16^*c*^	AMP, CIP, CST, NAL	CIP, LVX, CST	IncX4 (30)	4	CIP, NAL	

**Pig slaughterhouses and mWWTPs**									

05/01-09	*E. coli*	Influent in-house WWTP	8	AMP, CHL, CIP, CST, CTX, FOX, TET, TMP, FEP	PIP, TZP, CTX, CIP, LVX, CHL, SMX, CST	IncI1 (30)	8	TET	
05/01-21	*E. coli*	Influent in-house WWTP	8	AMP, CHL, CIP, CST, CTX, FOX, NAL, SMX, TET, TMP, FEP, ETP	PIP, TZP, CIP, LVX, CHL, SXT, CST	IncI1 (30)	8	TET	
05/01-22	*E. coli*	Influent in-house WWTP	4	AMP, CHL, CIP, CST, CTX, FOX, NAL, SMX, TET, TMP, FEP, ETP	PIP, TZP, CIP, LVX, CHL, SXT, CST	IncI1 (30)	4	TET	
05/01-23	*E. coli*	Influent in-house WWTP	4	AMP, CHL, CIP, CST, CTX, FOX, NAL, SMX, TET, TMP, FEP, ETP	PIP, TZP, CIP, LVX, CHL, SXT, CST	IncI1 (30)	4	TET	
05/02-28	*E. coli*	Effluent in-house WWTP	4	AMP, CHL, CIP, CST, CTX, FOX, NAL, SMX, TET, TMP, FEP, ETP	PIP, TZP, CIP, LVX, CHL, SXT, CST	IncI1 (30)	4	TET	
05/02-29	*E. coli*	Effluent in-house WWTP	4	AMP, CHL, CIP, CST, CTX, FOX, NAL, SMX, TET, TMP, FEP, ETP	PIP, TZP, CTX, CIP, LVX, CHL, SXT, CST	IncI1 (30)	4	TET	
05/06-69	*E. coli*	Aggregate wastewater from producing facilities	8	AMP, CHL, CIP, CST, CTX, FOX, NAL, SMX, TET, TMP, FEP, ETP	PIP, TZP, CIP, LVX, CHL, SXT, CST	IncI1 (30)	4	TET	
05/06-70	*E. coli*	Aggregate wastewater from producing facilities	8	AMP, CHL, CIP, CST, CTX, FOX, NAL, SMX, TET, TMP, FEP, ETP	PIP, TZP, CIP, LVX, CHL, SXT, CST	IncI1 (30)	4	TET	
C-03/01-04	*E. coli*	Influent in-house WWTP	8	AMP, CIP, CST, NAL, SMX, TET, TMP	PIP, CIP, LVX, CHL, CST	IncI1 (30)	4	CIP, NAL	
C-03/12-05	*E. coli*	On-site preflooder downstream	8	AMP, CHL, CIP, CST, CTX, FOX, NAL, SMX, CAZ, TET, FEP	PIP, CST	IncHI2 (n.d.*)	2	CIP, NAL	
C-03/12-07	*E. coli*	On-site preflooder downstream	4	AMP, CHL, CIP, CST, CTX, FOX, NAL, SMX, CAZ, TET, TGC, FEP	PIP, CST	IncHI2 (245)	2		

**Poultry slaughterhouses**									

C-03/12-08	*E. coli*	On-site preflooder downstream	8	AMP, CHL, CIP, CST, CTX, FOX, NAL, SMX, CAZ, TET, FEP	PIP, TZP, CST	IncHI2 (230)	4	CIP, NAL	
C-03/05-01	*K. pneumoniae*	Animal transporters	>64^*c*^	AMP, CHL, CIP, CST, NAL	PIP, CIP, LVX, CHL, CST	IncX4 (30)	4	CIP, NAL	
C-03/08-01	*K. pneumoniae*	Scalding water	>64^*c*^	AMP, CHL, CIP, CST, CTX, FOX, NAL, CAZ, FEP	FOF, CST	IncX4 (30)	4	CIP, NAL	

*^*a*^Antimicrobial Susceptibility Testing Plates of German Federal Institute for Risk Assessment (BfR) containing sulfamethoxazole (SMX), trimethoprim (TMP), chloramphenicol (CHL), ciprofloxacin (CIP), nalidixic acid (NAL), tetracycline (TET), tigecycline (TGC), ertapenem (ETP), meropenem (MEM), imipenem (IMI), cefotaxime (CTX), ceftazidime (CAZ), cefoxitin (FOX), cefepime (FEP), colistin (CST), ampicillin (AMP), gentamicin (GEN). MIC were interpreted according to the epidemiological cut off values of EUCAST. ^*b*^Micronaut-S MDR MRGN-Screening system containing temocillin (TEM), piperacillin (PIP), piperacillin/tazobactam (TZP), cefotaxime (CTX), ceftazidime (CAZ), imipenem (IMI), meropenem (MEM), amikacin (AMK), tigecycline (TGC), chloramphenicol (CHL), fosfomycin (FOF), trimethoprim/sulfamethoxazole (SXT), ciprofloxacin (CIP), levofloxacin (LVX), and colistin (CST). MIC were interpreted according to the clinical breakpoints of EUCAST. ^*c*^Polymorphisms found for coding sequences for PmrA or PmrB in 04/07-14: 157T→P (PmrB) and C-04/03-01: 73P→x (PmrB), 74S→x (PmrB). ^∗^n.d., not detected.*

**TABLE 3:** PmrAB polymorphisms of colistin-resistant *E. coli* isolates tested negative for *mcr-1* to *mcr-9*.

Isolate	Species	Origin	Colistin MIC, mg/L	Resistance phenotype (epidemiological cut-off values of EUCAST)^a^	Resistance phenotype (clinical breakpoints of EUCAST)^b^	PmrAB^*c*^
**Poultry slaughterhouses**						

C-01/07-07	*E. coli*	Stunning facilities	8	AMP, CIP, CST, NAL, SMX, TET	PIP, CST	2H→R (PmrB) 360A→V (PmrB)
C-01/07-08	*E. coli*	Stunning facilities	8	AMP, CIP, CST, NAL, SMX, TET	PIP, CST	2H→R (PmrB) 360A→V (PmrB)
C-01/07-11	*E. coli*	Stunning facilities	8	AMP, CIP, CST, NAL, SMX, TET	PIP, CST	2H→R (PmrB) 360A→V (PmrB)
C-01/07-12	*E. coli*	Stunning facilities	8	AMP, CIP, CST, NAL, SMX, TET	PIP, CST	2H→R (PmrB) 360A→V (PmrB)
04/02-15	*E. coli*	Transport crates	8	AMP, CHL, CIP, CST, CTX, NAL, SMX, CAZ, TET, TGC, FEP, FOX	PIP, CTX, CAZ, CIP, LVX, CHL, CST	−
04/02-16	*E. coli*	Transport crates	4	AMP, CST, CTX, SMX, CAZ, TET, TMP, FEP, FOX	PIP, CTX, CAZ, CIP, LVX, CHL, SXT, CST	−
04/07-11	*E. coli*	Stunning facilities	8	AMP, CHL, CIP, CST, CTX, NAL, SMX, CAZ, TET, FEP, FOX	PIP, CTX, CAZ, C/T, CIP, LVX, CHL, CST	−
C-04/03-08	*E. coli*	Scalding water	8	AMP, CST, SMX	PIP, CIP, LVX, SXT, CST	14L→P (PmrB) 44F→x (PmrB)
C-04/05-08	*E. coli*	Influent in-house WWTP	8	AMP, CST, SMX, TMP	PIP, SXT, CST	14L→P (PmrB) 44F→x (PmrB)
C-04/05-13	*E. coli*	Influent in-house WWTP	8	AMP, CST, SMX	PIP, CST	10L→R (PmrB) 12Q→x (PmrB)
C-04/05-15	*E. coli*	Influent in-house WWTP	8	AM, CIP, CST, NAL, TMP	PIP, CIP, LVX	15G→R (PmrA) 85T→A (PmrA) 2H→R (PmrB)
C-04/05-17	*E. coli*	Influent in-house WWTP	8	AMP, CIP, CST, NAL	PIP, CIP, LVX, CST	312D→N (PmrB)
C-04/07-03	*E. coli*	Stunning facilities	4	AMP, CHL, CIP, CST, CTX, NAL, SMX, CAZ, TET, FEP, FOX	PIP, CST	−
C-04/07-06	*E. coli*	Stunning facilities	4	AMP, CST, SMX, TET, TMP	PIP, CIP, LVX, SXT, FOF, CST	29S→x (PmrB)
C-04/07-08	*E. coli*	Stunning facilities	4	AMP, CST, SMX, TET, TMP	PIP, CIP, LVX, CHL, SXT, CST	−

**Pig slaughterhouses and mWWTPs**						

C-03/02-02	*E. coli*	Effluent in-house WWTP	8	AMP, CIP, CST, CTX, NAL, SMX, CAZ, TET, TMP, FEP	PP, CTX, CAZ, C/T, CIP, LVX, CHL, CST	204A→x (PmrA) 2H→R (PmrB)
C-03/10-10	*E. coli*	Influent municipal WWTP	4	CIP, CST, NAL	CST	80A→V (PmrA) 285A→T (PmrB) 333H→Q (PmrB)
C-03/10-14	*E. coli*	Influent municipal WWTP	4	CIP, CST, NAL	CST	80A→V (PmrA) 285A→T (PmrB) 333H→Q (PmrB)
03/10-43	*E. coli*	Influent municipal WWTP	16	AMP. CIP, CST, CTX, NAL, SMX, CAZ, TMP, FEP	PIP, CTX, CAZ, SXT, CST	44F→x (PmrB) 94P→S (PmrB)

*^*a*^Antimicrobial Susceptibility Testing Plates of German Federal Institute for Risk Assessment (BfR) containing sulfamethoxazole (SMX), trimethoprim (TMP), chloramphenicol (CHL), ciprofloxacin (CIP), nalidixic acid (NAL), tetracycline (TET), tigecycline (TGC), ertapenem (ETP), meropenem (MEM), imipenem (IMI), cefotaxime (CTX), ceftazidime (CAZ), cefoxitin (FOX), cefepime (FEP), colistin (CST), ampicillin (AMP), gentamicin (GEN). MIC were interpreted according to the epidemiological cut off values of EUCAST. ^*b*^Micronaut-S MDR MRGN-Screening system containing temocillin (TEM), piperacillin (PIP), piperacillin/tazobactam (TZP), cefotaxime (CTX), ceftazidime (CAZ), imipenem (IMI), meropenem (MEM), amikacin (AMK), tigecycline (TGC), chloramphenicol (CHL), fosfomycin (FOF), trimethoprim/sulfamethoxazole (SXT), ciprofloxacin (CIP), levofloxacin (LVX), and colistin (CST). MIC were interpreted according to the clinical breakpoints of EUCAST. ^*c*^Polymorphisms found for coding sequences for PmrA or PmrB.*

**TABLE 4:** PmrAB polymorphisms of colistin-resistant *K. pneumoniae* isolates tested negative for *mcr-1* to *mcr-9*.

Isolate	Species	Origin	Colistin MIC, mg/L	Resistance phenotype (epidemiological cut-off values of EUCAST)^a^	Resistance phenotype (clinical breakpoints of EUCAST)^b^	PmrAB^*c*^
**Poultry slaughterhouses**						
04/05-15	*K. pneumoniae*	Influent in-house WWTP	32	AMP, CIP, CST, CTX, NAL, SMX, CAZ, TMP, FEP	PIP, CTX, CAZ, CIP, LVX, CHL, SXT, FOF, CST	112T→P (PmrB)
04/70-34	*K. pneumoniae*	Stunning facilities	16	AMP, CHL, CST, CTX, SMX, CAZ, TET, TMP, FEP	CHL, SXT, FOF, CST	157T→P (PmrB)
C-04/02-07	*K. pneumoniae*	Transport crates	32	AMP, CIP, CST, NAL, SMX, TET, TMP	PIP, SXT, CST	
C-04/02-08	*K. pneumoniae*	Transport crates	>64	AMP, CIP, CST, NAL, SMX, CAZ, TET, TMP	PIP, SXT, CST	73P→x (PmrB) 74S→x (PmrB)
C-04/03-09	*K. pneumoniae*	Scalding water	32	AMP, CIP, CST, NAL, SMX, TET, TMP	PIP, CIP, LVX, SXT, CST	73P→x (PmrB) 74S→x (PmrB)
C-04/03-11	*K. pneumoniae*	Scalding water	32	AMP, CIP, CST, NAL, SMX, TET, TMP	PIP, CIP, LVX, SXT, FOF, CST	73P→x (PmrB) 74S→x (PmrB)
C-04/03-12	*K. pneumoniae*	Scalding water	32	AMP, CIP, CST, NAL, SMX, CAZ, TET, TGC, TMP, FEP, FOX	PIP, CIP, LVX, SXT, FOF, CST	73P→x (PmrB) 74S→x (PmrB)
C-04/03-13	*K. pneumoniae*	Scalding water	32	AMP, CIP, CST, NAL, SMX, TET, TMP	PIP, SXT, CST	73P→x (PmrB) 74S→x (PmrB)
C-04/05-01	*K. pneumoniae*	Influent in-house WWTP	32	AMP, CIP, CST, NAL, SMX, TET, TMP	PIP, SXT, CST	73P→x (PmrB) 74S→x (PmrB)
C-04/05-19	*K. pneumoniae*	Influent in-house WWTP	32	AMP, CIP, CST, NAL, SMX, TET, TMP	PIP, CIP, LVX, SXT, FOF, CST	73P→x (PmrB) 74S→x (PmrB)
C-04/07-29	*K. pneumoniae*	Stunning facilities	32	AMP, CIP, CST, NAL, SMX, TET, TMP	PIP, SXT, COL	73P→x (PmrB) 74S→x (PmrB)
C-04/07-02	*K. pneumoniae*	Stunning facilities	>64	AMP, CIP, CST, NAL, SMX	CST	
C-04/07-25	*K. pneumoniae*	Stunning facilities	32	AMP, CIP, CST, NAL, SMX, TET, TMP	PIP, CST	73P→x (PmrB) 74S→x (PmrB)
C-04/07-28	*K. pneumoniae*	Stunning facilities	32	AMP, CIP, CST, CTX, NAL, TET, TGC, FEP, FOX	PIP, CST	73P→x (PmrB) 74S→x (PmrB)


**Pig slaughterhouses and mWWTPs**						

C-03/08-02	*K. pneumoniae*	Scalding water	16	AMP, CST, SMX, CAZ, TET, FEP	PIP, TZP, C/T, CST	
C-03/10-18	*K. pneumoniae*	Influent municipal WWTP	16	AMP, CST	FOF, CST	217A→V (PmrA)
C-03/10-19	*K. pneumoniae*	Influent municipal WWTP	16	AMP, CST	FOF, CST	217A→V (PmrA)
C-03/10-21	*K. pneumoniae*	Influent municipal WWTP	32	AMP, CST	PIP, FOF, CST	2A→S (PmrB)
C-03/10-22	*K. pneumoniae*	Influent municipal WWTP	32	AMP, CST	CST	2A→S (PmrB)
C-03/12-01	*K. pneumoniae*	On-site preflooder downstream	8	AMP, CIP, CST, NAL, CAZ, FEP	PIP, CTX, CAZ, C/T, CIP, LVX, CHL, CST	“Insertion” of QLQQLARVG between 201E and 202Q
C-03/12-02	*K. pneumoniae*	On-site preflooder downstream	16	AMP, CIP, CST, NAL, SMX, CAZ, TET, FEP	PIP, CTX, CAZ, C/T, CIP, LVX, CHL, CST	
C-03/12-06	*K. pneumoniae*	On-site preflooder downstream	16	AMP, CHL, CIP, CST, NAL, SMX, CAZ, TET, FEP	PIP, CTX, CAZ, C/T, CIP, LVX, CHL, CST	
C-03/12-10	*K. pneumoniae*	On-site preflooder downstream	>64	AMP, CHL, CIP, CST, NAL	PIP, CTX, CAZ, CIP, LVX, CHL, CST	217A→V (PmrA)
C-05/10-15	*K. pneumoniae*	Influent municipal WWTP	16	AMP, CST	PIP, CST	147A→E (PmrA) 217A→V (PmrA)
C-05/10-16	*K. pneumoniae*	Influent municipal WWTP	16	AMP, CST	CST	147A→E (PmrA) 217A→V (PmrA)
C-05/10-26	*K. pneumoniae*	Influent municipal WWTP	16	AMP, CST	FOF, CST	37A→T (PmrA)
						

**Poultry slaughterhouses**						

05/11-29	*K. pneumoniae*	Effluent municipal WWTP	32	AMP, CHL, CIP, CST, CTX, NAL, CAZ, FEP, ETP, FOX, IMI	PIP, TZP, CTX, CAZ, CIP, LVX, CHL, FOF, CST	57E→G (PmrA) 203S→P (PmrB)

*^*a*^Antimicrobial Susceptibility Testing Plates of German Federal Institute for Risk Assessment (BfR) containing sulfamethoxazole (SMX), trimethoprim (TMP), chloramphenicol (CHL), ciprofloxacin (CIP), nalidixic acid (NAL), tetracycline (TET), tigecycline (TGC), ertapenem (ETP), meropenem (MEM), imipenem (IMI), cefotaxime (CTX), ceftazidime (CAZ), cefoxitin (FOX), cefepime (FEP), colistin (CST), ampicillin (AMP), gentamicin (GEN). MIC were interpreted according to the epidemiological cut off values of EUCAST. ^*b*^Micronaut-S MDR MRGN-Screening system containing temocillin (TEM), piperacillin (PIP), piperacillin/tazobactam (TZP), cefotaxime (CTX), ceftazidime (CAZ), imipenem (IMI), meropenem (MEM), amikacin (AMK), tigecycline (TGC), chloramphenicol (CHL), fosfomycin (FOF), trimethoprim/sulfamethoxazole (SXT), ciprofloxacin (CIP), levofloxacin (LVX), and colistin (CST). MIC were interpreted according to the clinical breakpoints of EUCAST. ^*c*^Polymorphisms found for coding sequences for PmrA or PmrB.*

According to the scheme B, the isolates with exception of *K. pneumoniae* had lower resistance rates to 3rd generation cephalosporins (CTX and CAZ). The differences varied between 12.3% for CAZ by *E. coli* and 26.6% for CTX by *E. cloacae* complex (Figures 4, 5). Furthermore, they were susceptible to temocillin, ceftazidime-avibactam, imipenem, meropenem, amikacin and, with exception of some *E. cloacae* complex isolates, to tigecycline.

The highest 3MDRO rates (multidrug-resistant organisms with combined resistance to PIP, CTX, and CIP) were exhibited by *K. pneumoniae* (26.5%), followed by *E. cloacae* complex (20.6%) and *E. coli* (13.8%). However, if using piperacillin-tazobactam instead of piperacillin for determination of the MDR status, as recommended by Magiorakos et al. (2012), the 3MDRO rates were lower at 5.9% for *K. pneumoniae*, 3.3% for *E. cloacae* complex, and 3.1% for *E. coli*.

### Phylogenetic Groups of *E. coli* (*n* = 65)

The majority of the *E. coli* isolates belonged to the most common phylogroups associated with commensal strains, such as A (32.3%), B1 (24.6%), C (16.9%), F (10.8%), Clade I, II (9.2%), and E (1.5%) (Clermont et al., 2000, 2013). Only two isolates (3.0%) recovered from the influent of the in-house WWTP of a poultry slaughterhouse were assigned to extraintestinal pathogenic (ExPEC) group D (Clermont et al., 2000, 2013). Furthermore, one isolate originating from the wastewater used for cleaning of poultry stunning facilities belonged to group B2.

### Occurrence of *mcr* Genes

Of the *mcr* genes screened, only *mcr-1.1* was detected in 70.8% of *E. coli* and 20.6% of *K. pneumoniae* isolates. Colistin MICs of *mcr-1*-positive *E. coli* isolates ranged from 4 to 8 mg/L, whereas *mcr-1* carrying *K. pneumoniae* isolates expressed higher level of resistance from 4 to >64 mg/L.

In poultry and pig slaughterhouses the *mcr-1.1* carrying isolates of *E. coli* and *K. pneumoniae* were detected at almost all sampling points including scalding water and effluents of the in-house WWTPs. Furthermore, *mcr-1.1* positive isolates of *E. coli* were detected in on-site preflooders downstream the discharge point. Detailed information on the isolation source and phenotypic resistance of *mcr-1.1* carrying isolates of *E. coli* and *K. pneumoniae* is given in Table 2.

### PFGE Patterns of Colistin-Resistant *mcr-1* Carrying Isolates, Location of *mcr-1* Gene

Overall, the analyzed isolates (*n* = 53, 46 *E. coli* and 7 *K. pneumoniae*) exhibited a broad diversity as they were assigned to 25 different *Xba*I profiles (20 for *E. coli* and 5 for *K. pneumoniae*). S1 nuclease PFGE, followed by Southern blot hybridization revealed the presence of *mcr-1* carrying plasmids ranging between 30 and 360 kb. Interestingly, the majority of the isolates exhibited a predominant plasmid type of 30 kb (Table 2). However, we had also determined a substantial number of isolates exhibiting the same *Xba*I macrorestriction patterns and/or plasmid profiles.

### Conjugation and Transformation Experiments, Inc-Typing of Plasmids

In 67.4% (31/46) of *mcr-1* carrying *E. coli* isolates, the *mcr-1* gene was found to be encoded on plasmids of different Inc-groups that could be conjugated into recipient *E. coli* cells (Table 1). Plasmids were affiliated to IncI1 (41.9%), IncHI2, and IncX4 (each 22.6%), IncF (9.7%) as well as IncI2 (3.2%) types as demonstrated by TaqMan RT-PCR and PBRT method. All seven *mcr-1*-positive *K. pneumoniae* isolates carried the *mcr-1* on self-transmissible IncX4 (71.4%) and IncI1 (28.6%) plasmids. Of note, IncI1-type plasmids carrying *mcr-1* were predominant in all sampling sites. Colistin MICs of transconjugants were either identical or lower than those of the donor strains and ranged from 2 to 8 mg/L.

Conjugation experiments with the applied selection conditions resulted in diverse co-transferred resistance phenotypes. Using epidemiological cut-off values, 81.6% (31/38) of *E. coli* and *K. pneumoniae* transconjugants expressed resistance to further antimicrobials beside colistin. Among the isolates recovered in the poultry slaughterhouses the most frequently co-transferred resistance was to ciprofloxacin and nalidixic acid (70.8%, 17/24), followed by ampicillin (29.2%, 7/24) and trimethoprim/sulfamethoxazole (25.0%, 6/24). Only 8.3% (2/24) of the isolates co-transferred resistance against 3rd generation cephalosporins. In contrast, the majority of the isolates originating from the pig slaughterhouses co-transferred resistance against tetracycline (57.1%, 8/14). The resistance to ciprofloxacin and nalidixic acid was co-transferred by 35.7% (5/14) of the isolates. However, when applying scheme B based on clinical breakpoints, only 15.8% (6/38) of the colistin-resistant transconjugants expressed additional resistances, mostly to sulfamethoxazole-trimethoprim (5/6) and piperacillin (4/6).

The transformation experiments with 15 *E. coli* isolates carrying *mcr-1* gene (15/46) and for which no transconjugants could be obtained, did not yield any transformants.

Detailed information on Inc-types of *mcr-1* harboring plasmids, colistin MIC of the transconjugants and co-transferred resistance phenotypes of individual isolates is given in Table 2.

### pmrAB

Sequences of Colistin-Resistant *E. coli* and *K. pneumoniae* Isolates Tested Negative for *mcr-1* to *mcr-9*

In 73.7% (14/19) of *E. coli* isolates non-synonymous polymorphisms at the protein level were detected in *pmrA* and *pmrB*. Nucleotide sequence polymorphisms that produce protein variants 15Gly→Arg, 80Ala→Val, 85Thr→Ala, 204Ala→X were found in *pmrA*. Furthermore, eleven variants, 2His→Arg, 10Leu→Arg, 12Gln→x, 14Leu→Pro, 29Ser→x, 44Phe→x, 94Pro→S, 285Ala→Thr, 312Asp→Asn, 333His→Gln, 360Ala→Val, were found in *pmrB*.

In 81.5% (22/27) of *K. pneumoniae* isolates the *pmrA* and *pmrB* genes revealed polymorphic positions that were non-synonymous at the protein level. Additionally, four non-synonymous polymorphisms were found in *pmrA* (37Ala→Thr, 57Glu→Gly, 147Ala→Glu, and 217Ala→Val) and six in *pmrB* (2Ala→Ser, 73Pro→x, 74Ser→x, 112Thr→Pro, 157Thr→Pro, 203Ser→Pro). In one *K. pneumoniae* isolate recovered from the on-site preflooder downstream the discharge point, a yet unknown insertion of nine amino acids (Gln-Leu-Gln-Gln-Leu-Ala-Arg-Val-Gly) between amino acid residues Glu-201 and Gln-202 of *pmrB* was identified. Detailed information on non-synonymous polymorphisms of individual *E. coli* and *K. pneumoniae* isolates, their origin and resistance phenotypes is given in Tables 3, 4, respectively.

## Discussion

Our study provides data on the occurrence of colistin resistant *Enterobacteriaceae* (*E. coli*, *K. pneumoniae*, and *E. cloacae* complex) in process waters and wastewater along the slaughtering processes in poultry and pig slaughterhouses, their in-house and mWWTPs as well as receiving waterbodies.

The highest prevalence of colistin-resistant bacteria was detected in poultry slaughterhouses. This is in accordance with other studies indicating frequent occurrence of colistin-resistant *Enterobacteriaceae* in the poultry production chain in Germany (Irrgang et al., 2016; Inderbinen, 2017). Current data from official bodies on antimicrobial usage in different animal species in Germany are not available. However, the Report of the Federal Ministry of Food and Agriculture on the Evaluation of the Antimicrobials Minimization Concept introduced with the 16th Act to Amend the Medicinal Products Act (16th AMG Amendment) indicates a higher usage of colistin in German poultry production in comparison to other livestock production chains (BMEL, 2019). Moreover, between 2014 and 2017 consumption of polypeptide antibiotics in broiler production in Germany slightly increased from 11 to 13 tons. Whereas in pig production chain polypeptide antibiotics are mostly used to treat piglets and for the treatment of fattening pigs a decrease from 4 tons in 2014 to 0.5 tons in 2017 was observed (BMEL, 2019). Thus, the higher use of colistin in poultry may coincide with the frequent occurrence of colistin-resistant bacteria in this production chain. Furthermore, in comparison to poultry, a longer life span and time gap between administration of antibiotic and slaughtering among pigs may result in a decrease of colistin resistance when selection pressure is absent. Moreover, the kind of antibiotic treatment, e.g., treatment of individual pigs or small groups thereof in comparison to the whole flock treatment, may also be responsible for the lower occurrence of colistin resistance among pigs and accordingly in the pig slaughterhouses (BMEL, 2010). Furthermore, our results are in line with the EU summary report on Antimicrobial Resistance in zoonotic and indicator bacteria from humans, animals and food in 2017/2018 (ECDC, 2020) showing increased colistin resistance in *E. coli* isolates from broilers compared to those from pigs.

From nine *mcr* genes tested, *mcr-1* was the most prevalent one, which corroborates the study of Elbediwi et al. (2019) that emphasizes the global dissemination and high prevalence of *mcr-1* gene among colistin-resistant bacteria isolated from animals and food products worldwide. With prevalences of 0.04 to 20.3%, *mcr-1* is predominantly detected in *Enterobacteriaceae* isolates (*E. coli*, *Klebsiella* spp., *Enterobacter* spp., *Salmonella* spp., and *Shigella* spp.) from livestock, retail meat (1.4–19%) and to a lesser extent in human clinical isolates (0.06–2%), worldwide (Hasman et al., 2015; Haenni et al., 2016; Kluytmans-van, den Bergh et al., 2016; Malhotra-Kumar et al., 2016; Webb et al., 2016). In Germany, colistin-resistant isolates from turkey and broilers food chains show the highest *mcr-1* prevalence in comparison to pigs and cattle (Irrgang et al., 2016; Borowiak et al., 2020). Thus, livestock and poultry are considered as an origin of *mcr-1* and is its important reservoir for transmission to humans (Liu et al., 2016). Based on the wide dissemination of *mcr-1*, EMA’s (European Medicines Agency) Antimicrobial Advice *Ad Hoc* Expert Group (AMEG) advised to minimize sales of colistin for use in animals EU-wide to achieve a 65% reduction in 2016 (EMA, 2016).

The genes *mcr-2* to *mcr-9* have not been detected in our study. This could be due to their limited geographical distribution and bacterial host range (Borowiak et al., 2020) as well as substantially low prevalence compared with *mcr-1*. While *mcr-2* to *mcr-8* are being detected mostly in *E. coli* and *K. pneumoniae* isolates from pigs and poultry in China and South Europe (Xavier et al., 2016; AbuOun et al., 2017; Borowiak et al., 2017; Carattoli et al., 2017; Yin et al., 2017; Wang et al., 2018; Yang et al., 2018), *mcr-9* and *mcr-10* were discovered in clinical strains of *Salmonella enterica* serotype Typhimurium (Carroll et al., 2019) and *Enterobacter roggenkampii*, respectively (Wang et al., 2020). Currently in Germany, *mcr-3* was detected in *Aeromonas* spp. isolates of fish origin (Eichhorn et al., 2018). Furthermore, *mcr-4* has been frequently identified in different *Salmonella* serovars from poultry meat and pork (Borowiak et al., 2020) as well as *mcr-5* has been detected in *E. coli* and *Salmonella* isolates of livestock origin (Hammerl et al., 2018; Borowiak et al., 2020). Kneis et al. (2019) reported high abundances of *mcr-3*, *mcr-4*, *mcr-5*, and *mcr-7* in German mWWTPs. However, the possible origin sources such as households, health care facilities, livestock farming sites or slaughterhouses as well as bacterial host ranges have not been identified (Kneis et al., 2019).

*Escherichia coli* isolates carrying *mcr-1* on transferable IncHI2 plasmids were detected in on-site preflooder downstream the discharge point of mWWTP. Possible entry sources could be run-offs from the fields fertilized with contaminated manure (Guenther et al., 2017) and feces of wild animals (birds) (Lin et al., 2020). Previously, (Zurfluh et al., 2016) and (Falgenhauer et al., 2019) detected *mcr-1* harboring *E. coli* in surface water and rivers in Switzerland and Germany, respectively. Moreover, in our study *mcr-1*-positive *K. pneumoniae* was recovered from poultry scalding water. This could be a possible source of contamination of carcasses and products and lead to the introduction of *mcr-1* carrying *K. pneumoniae* into the food chain. (Schrauwen et al., 2017) reported that 24.8% of retail chicken meat in Netherlands were positive for *mcr-1*, carried mostly by *E. coli* and to a lesser extent by *K. pneumoniae*. Furthermore, 40.6% of poultry meat samples originating from Germany were contaminated with *mcr-1* producing bacteria (Inderbinen, 2017). Some of the *mcr-1* carrying isolates recovered from wastewater used for cleaning of stunning facilities and influents of in-house WWTP from poultry slaughterhouses belonged to ExPEC groups B2 and D, which are known to harbor more virulence factors than commensal strains and pose a zoonotic risk (Johnson et al., 2012). This enables the transmission of *mcr-1*-positive ExPECs of poultry origin to humans and represents a potential vehicle of *mcr* genes for human diseases, e.g., bloodstream and urinary tract infections (Izdebski et al., 2016; Zhong et al., 2019). Moreover, study of Zhuge et al. (2019) shown that *mcr-1*-positive *E. coli* of phylogroups B1 and F also possessed high virulence in rodent models for ExPEC-associated human infections and could therefore pose an elevated risk of infections for humans.

According to the classification of Magiorakos et al. (2012) and applying epidemiological cut-off values, target isolates showed high percentage of multidrug resistance (combined resistance to CST, CIP, and TET) with the highest rate of 49.2% for *E. coli*. However, it is important to note that from a human clinical perspective, the antibiotic groups are not considered to be equally clinically relevant (Exner et al., 2017). Thus, from the point of view of KRINKO, the multidrug resistance rates (3MDRO rates, combined resistance to TZP, CTX, and CIP) were low with the highest percentage of 5.9% for *K. pneumoniae*. For this evaluation the combination of piperacillin/tazobactam instead of piperacillin is used, as in the clinical practice in Germany piperacillin is administered only in combination with β-lactamase inhibitors. Furthermore, applying clinical breakpoints, isolates were completely susceptible to reserve antibiotics ceftazidime-avibactam and tigecycline as well as carbapenems (IMP and MEM). Moreover, temocillin, which was introduced in 2019 for therapy of extended spectrum β-lactamase (ESBL) and AmpC producers, and amikacin, classified by WHO as reserve second−line drug, were also effective against all isolates. Thus, these substances could be still effective in antimicrobial therapy in case of infection.

It was already reported that *mcr-1* gene occurs frequently in isolates that are susceptible to most classes of antimicrobials (EMA, 2016). However, this contrasts with the findings of our study, as considerable percentage of colistin-resistant *mcr-1* positive isolates from our study showed resistance of up to eleven antibiotics, including clinically relevant ones. This reinforces the theory that possible transmission of *mcr-1* gene to highly virulent bacteria carrying other antimicrobial resistance genes, e.g., ESBL or carbapenemases would narrow clinical therapeutic options (Forde et al., 2018). Zheng et al. (2017) and Zheng et al. (2016) reported on *E. coli* isolates from blood stream infections which co-produce NDM-1 and MCR-1.

In our study *mcr-1* gene was detected in a wide range of plasmid types such as IncI1, IncHI2, IncX4, IncF, and IncI2, which is in consent with other reports. *mcr-1* located on IncI1 plasmids was detected in *E. coli* recovered from pig manure (Guenther et al., 2017) and chicken feces (Hassen et al., 2020). *E. coli* isolates recovered from pigs in Portugal carried *mcr-1* on IncHI2 and IncX4 plasmids (Kieffer et al., 2017). In another study, *mcr-1* gene was located on IncX4 and IncHI2 plasmids in *E. coli* from broilers and veal calves in Netherlands (Veldman et al., 2016). Furthermore, Gelbíčová et al. (2019) isolated *E. coli*, *K. pneumoniae*, and *Citrobacter braakii* from raw turkey meat and liver which harbored *mcr-1* gene on IncX4, IncHI2, and IncI2 plasmids. In addition to livestock and food products, MCR-1-producing *E. coli* which carry the resistance on IncX4, IncHI2, and IncI1 types of plasmids, were isolated from different environmental sources such as surface water in Germany (Falgenhauer et al., 2019) and public seawater beach in Norway (Jørgensen et al., 2017). The association of *mcr-1* gene with insertion sequence IS*Apl1* might play a major role in its mobilization, its further successful establishment in BHR plasmids and subsequent dissemination among *Enterobacteriaceae* (Snesrud et al., 2016; Poirel et al., 2017). On the other hand, without colistin exposure, IS*Apl1* is able to facilitate the deletion of resistance genes, as described by Zhang et al. (2019) for *mcr-1* and *mcr-3.19.*

The co-transfer of the decreased susceptibility to fluoroquinolones (MIC of CIP 0.25 mg/L) by the majority of the isolates recovered in the poultry slaughterhouses could be due to plasmid-mediated quinolone resistance (PMQR) genes. They are known to provide only low-level resistance that by itself does not exceed the clinical breakpoint of >0.5 mg/L for susceptibility (Jacoby et al., 2014). Furthermore, resistance to tetracyclines was co-transferred by the isolates from pig slaughterhouses, as tetracycline resistance genes are often located on mobile genetic elements such as plasmids, transposons, conjugative transposons, and/or integrons (Roberts, 2003). Thus, fluoroquinolones and tetracyclines, which make up 25.7% of the total antimicrobial usage in the veterinary medicine in Germany (BVL, 2019), may impose a selective pressure that could favor the selection of *mcr* genes, even without use of colistin and vice versa. Moreover, Savin et al. (2020a) reported on antimicrobial residues of ampicillin, ciprofloxacin, and ofloxacin detected in German mWWTPs which exceeded their PNECs (Predicted No Effect Concentration) (Bengtsson-Palme and Larsson, 2016). Ofloxacin exceeded its PNEC even after dilution of the treated wastewater with the recipient water. This may contribute to the co-selection of *mcr-1* carrying bacteria in surface water, whereas the residues of ampicillin may promote the dissemination of *mcr-*carrying strains of species with intrinsic resistance to this antimicrobial (e.g., *Klebsiella* spp., *E. cloacae* complex).

The great majority of colistin-resistant *E. coli* and *K. pneumoniae* which were tested negative for known *mcr* genes harbored chromosomal point mutations in the *pmrAB* coding regions. For *E. coli*, a mutation at the amino acid position 10 in *pmrB* has been detected by Cannatelli et al. (2017) leading to the substitution 10Leu→Pro that confers resistance to colistin. However, in our study, the polymorphisms at this position resulted in leucine to arginine substitution. One *K. pneumoniae* isolate recovered from the wastewater used for cleaning of poultry stunning facilities demonstrated mutation 157T→P (PmrB) that has been previously reported in *K. pneumoniae* from patients and healthy humans (Olaitan et al., 2014) as well as in clinical colistin-resistant *K. pneumoniae* carbapenemase (KPC)-producing isolates (Leung et al., 2017). Furthermore, a substitution 217A→V (PmrA) that has been already described in colistin-resistant isolates from clinical blood cultures (Esposito et al., 2018) was found in isolates recovered from the influent of mWWTPs and on-site preflooders. To determine whether other detected polymorphisms in *E. coli* and *K. pneumoniae* cause resistance to colistin, complementation assays are needed.

We are not aware of other studies in Germany that investigated such environmental samples (i.e., process waters and wastewater) which have been taken directly in the slaughterhouses and their on-site WWTPs that underlines the novelty of our study. In conclusion, our results indicate high prevalence of *E. coli* isolates which carry *mcr-1* on a wide variety of transferable plasmids in process water accruing along the slaughtering process in German poultry slaughterhouses. This may pose an elevated risk of colonization for slaughterhouse employees with occupational exposure to process water and wastewater. Furthermore, despite strict hygiene rules established in German slaughterhouses, *mcr-1* carrying bacteria could be introduced into the food chain through cross-contamination (e.g., scalding water). Moreover, due to insufficient treatment of wastewater, such strains were discharged into the environment. In order to determine the persistence of *mcr-1* carrying *E. coli* isolates in the receiving water bodies, further investigations are needed. Furthermore, besides colistin, overall reduction of the use of antibiotics in livestock is required, as it was shown that *mcr-1* can be also co-selected by fluoroquinolones and tetracyclines. In this way, the input of resistant bacteria into the slaughterhouses can be reduced. Additionally, as *mcr-1* carrying isolates were detected in the effluent of the WWTPs, a broad dissemination the environment can be expected. Thus, this study supports the necessity of the implementing of advanced wastewater treatment technologies to limit the exposition of the environment with bacteria expressing resistances against last resort antimicrobials.

## Data Availability Statement

The raw data supporting the conclusions of this article will be made available by the authors, without undue reservation.

## Author Contributions

MS: project administration, conceptualization, methodology, investigation, writing – original draft, and visualization. GB, KS, and RS: writing – review and editing. KB, CH, and JH: investigation, writing – review and editing. MP and ES: investigation. JK: conceptualization, writing – review and editing, supervision, and funding acquisition. All authors contributed to the article and approved the submitted version.

## Conflict of Interest

The authors declare that the research was conducted in the absence of any commercial or financial relationships that could be construed as a potential conflict of interest.

## References

[B1] AbuOun M. Stubberfield E. J. Duggett N. A. Kirchner M. Dormer L. Nunez-Garcia J. (2017). *mcr-1* and *mcr-2* variant genes identified in *Moraxella* species isolated from pigs in Great Britain from 2014 to 2015. *J. Antimicrob. Chemother.* 72 2745–2749. 10.1093/jac/dkx286 29091227 PMC5890717

[B2] Aldous W. K. Pounder J. I. Cloud J. L. Woods G. L. (2005). Comparison of six methods of extracting *Mycobacterium tuberculosis* DNA from processed sputum for testing by quantitative real-time PCR. *J. Clin. Microbiol.* 43 2471–2473. 10.1128/JCM.43.5.2471-2473.2005 15872286 PMC1153782

[B3] Azzopardi E. A. Boyce D. E. Thomas D. W. Dickson W. A. (2013). Colistin in burn intensive care: back to the future? *Burns* 39 7–15. 10.1016/j.burns.2012.07.015 22871554

[B4] Bengtsson-Palme J. Larsson D. G. J. (2016). Concentrations of antibiotics predicted to select for resistant bacteria: proposed limits for environmental regulation. *Environ. Int.* 86 140–149. 10.1016/j.envint.2015.10.015 26590482

[B5] Blau K. Bettermann A. Jechalke S. Fornefeld E. Vanrobaeys Y. Stalder T. (2018). The transferable resistome of produce. *mBio* 9:e01300-18. 10.1128/mBio.01300-18 30401772 PMC6222124

[B6] BMEL (2010). *Guidelines for the Prudent Use of Veterinary Antimicrobial Drugs -with Notes for Guidance.* Bonn: Federal Ministry of Food and Agriculture.

[B7] BMEL (2019). *Report of the Federal Ministry of Food and Agriculture on the Evaluation of the Antibiotics Minimisation Concept Introduced with the 16th Act to Amend the Medicinal Products Act (16th AMG Amendment): Evaluation Based on Section 58g of the Medicinal Products Act.* Bonn: Federal Ministry of Food and Agriculture.

[B8] Borowiak M. Baumann B. Fischer J. Thomas K. Deneke C. Hammerl J. A. (2020). Development of a novel *mcr-6* to *mcr-9* multiplex PCR and assessment of *mcr-1* to *mcr-9* occurrence in colistin-resistant *Salmonella enterica* isolates from environment, feed, animals and food (2011-2018) in Germany. *Front. Microbiol.* 11:80. 10.3389/fmicb.2020.00080 32117115 PMC7011100

[B9] Borowiak M. Fischer J. Hammerl J. A. Hendriksen R. S. Szabo I. Malorny B. (2017). Identification of a novel transposon-associated phosphoethanolamine transferase gene, *mcr-5*, conferring colistin resistance in d-tartrate fermenting *Salmonella enterica* subsp. *enterica* serovar paratyphi B. *J. Antimicrob. Chemother.* 72 3317–3324. 10.1093/jac/dkx327 28962028

[B10] BVL (2019). *Vergleich der Antibiotika-Abgabemengen Bezogen auf die Wirkstoffklassen 2011 bis 2018.* Berlin: Bundesamt fur Verbraucherschutz und Lebensmittelsicherheit.

[B11] Cannatelli A. Giani T. Aiezza N. Di Pilato V. Principe L. Luzzaro F. (2017). An allelic variant of the PmrB sensor kinase responsible for colistin resistance in an *Escherichia coli* strain of clinical origin. *Sci. Rep.* 7:5071. 10.1038/s41598-017-05167-6 28698568 PMC5506025

[B12] Carattoli A. Bertini A. Villa L. Falbo V. Hopkins K. L. Threlfall E. J. (2005). Identification of plasmids by PCR-based replicon typing. *J. Microbiol. Methods* 63 219–228. 10.1016/j.mimet.2005.03.018 15935499

[B13] Carattoli A. Villa L. Feudi C. Curcio L. Orsini S. Luppi A. (2017). Novel plasmid-mediated colistin resistance *mcr-4* gene in *Salmonella* and *Escherichia coli*, Italy 2013, Spain and Belgium, 2015 to 2016. *Euro Surveill.* 22:30589. 10.2807/1560-7917.ES.2017.22.31.30589 28797329 PMC5553062

[B14] Carroll L. M. Gaballa A. Guldimann C. Sullivan G. Henderson L. O. Wiedmann M. (2019). Identification of novel mobilized colistin resistance gene *mcr-9* in a multidrug-resistant, colistin-susceptible *Salmonella enterica* serotype Typhimurium isolate. *mBio* 10:e00853-19. 10.1128/mBio.00853-19 31064835 PMC6509194

[B15] CDC (2020). *Standard Operating Procedure for PulseNet PFGE of Escherichia coli O157:H7, Escherichia coli non-O157 (STEC), Salmonella serotypes, Shigella sonnei and Shigella flexneri.* Available online at: https://www.cdc.gov/pulsenet/pdf/ecoli-shigella-salmonella-pfge-protocol-508c.pdf (accessed August 29, 2020).

[B16] Clermont O. Bonacorsi S. Bingen E. (2000). Rapid and simple determination of the *Escherichia coli* phylogenetic group. *Appl. Environ. Microbiol.* 66 4555–4558. 10.1128/aem.66.10.4555-4558.2000 11010916 PMC92342

[B17] Clermont O. Christenson J. K. Denamur E. Gordon D. M. (2013). The Clermont *Escherichia coli* phylo-typing method revisited: improvement of specificity and detection of new phylo-groups. *Environ. Microbiol. Rep.* 5 58–65. 10.1111/1758-2229.12019 23757131

[B18] Dohmen W. van Gompel L. Schmitt H. Liakopoulos A. Heres L. Urlings B. A. (2017). ESBL carriage in pig slaughterhouse workers is associated with occupational exposure. *Epidemiol. Infect.* 145 2003–2010. 10.1017/S0950268817000784 28462735 PMC9203444

[B19] ECDC (2020). The European Union summary report on antimicrobial resistance in zoonotic and indicator bacteria from humans, animals and food in 2017/2018. *EFSA J.* 18:6007. 10.2903/j.efsa.2020.6007 32874244 PMC7448042

[B20] Eichhorn I. Feudi C. Wang Y. Kaspar H. Feßler A. T. Lübke-Becker A. (2018). Identification of novel variants of the colistin resistance gene mcr-3 in *Aeromonas* spp. from the national resistance monitoring programme GERM-Vet and from diagnostic submissions. *J. Antimicrob. Chemother.* 73 1217–1221. 10.1093/jac/dkx538 29394397

[B21] Elbediwi M. Li Y. Paudyal N. Pan H. Li X. Xie S. (2019). Global burden of colistin-resistant bacteria: mobilized colistin resistance genes study (1980-2018). *Microorganisms* 7:461. 10.3390/microorganisms7100461 31623244 PMC6843232

[B22] EMA (2016). *Updated Advice on the Use of Colistin Products in Animals within the European Union: Development of Resistance and Possible Impact on Human and Animal Health (EMA/CVMP/CHMP/231573/2016).* Amsterdam: EMA.

[B23] EMEA (2002). *Committee for Veterinary Medicinal Products. Colistin. Summary Report (2), EMEA/MRL/815/02-FINAL*. Available online at: https://www.ema.europa.eu/en/documents/mrl-report/colistin-summary-report-2-committee-veterinary-medicinal-products_en.pdf

[B24] Esposito E. P. Cervoni M. Bernardo M. Crivaro V. Cuccurullo S. Imperi F. (2018). Molecular epidemiology and virulence profiles of colistin-resistant *Klebsiella pneumoniae* blood isolates from the hospital agency “Ospedale dei Colli,” Naples, Italy. *Front. Microbiol.* 9:1463. 10.3389/fmicb.2018.01463 30061868 PMC6054975

[B25] Exner M. Bhattacharya S. Christiansen B. Gebel J. Goroncy-Bermes P. Hartemann P. (2017). Antibiotic resistance: what is so special about multidrug-resistant Gram-negative bacteria? *GMS Hyg. Infect. Control* 12:Doc05. 10.3205/dgkh000290 28451516 PMC5388835

[B26] Falagas M. E. Kasiakou S. K. (2005). Colistin: the revival of polymyxins for the management of multidrug-resistant gram-negative bacterial infections. *Clin. Infect. Dis.* 40 1333–1341. 10.1086/429323 15825037

[B27] Falgenhauer L. Schwengers O. Schmiedel J. Baars C. Lambrecht O. Heß S. (2019). Multidrug-resistant and clinically relevant gram-negative bacteria are present in German surface waters. *Front. Microbiol.* 10:2779. 10.3389/fmicb.2019.02779 31849911 PMC6896662

[B28] Forde B. M. Zowawi H. M. Harris P. N. A. Roberts L. Ibrahim E. Shaikh N. (2018). Discovery of *mcr-1*-mediated colistin resistance in a highly virulent *Escherichia coli* lineage. *mSphere* 3:e00486-18. 10.1128/mSphere.00486-18 30305321 PMC6180223

[B29] Gelbíčová T. Baráková A. Florianová M. Jamborová I. Zelendová M. Pospíšilová L. (2019). Dissemination and comparison of genetic determinants of mcr-mediated colistin resistance in *Enterobacteriaceae* via retailed raw meat products. *Front. Microbiol.* 10:2824. 10.3389/fmicb.2019.02824 31921017 PMC6920100

[B30] Guenther S. Falgenhauer L. Semmler T. Imirzalioglu C. Chakraborty T. Roesler U. (2017). Environmental emission of multiresistant *Escherichia coli* carrying the colistin resistance gene *mcr-1* from German swine farms. *J. Antimicrob. Chemother.* 72 1289–1292. 10.1093/jac/dkw585 28122910

[B31] Hadjadj L. Riziki T. Zhu Y. Li J. Diene S. M. Rolain J.-M. (2017). Study of *mcr-1* gene-mediated colistin resistance in *Enterobacteriaceae* isolated from humans and animals in different countries. *Genes* 8:394. 10.3390/genes8120394 29257080 PMC5748712

[B32] Haeili M. Javani A. Moradi J. Jafari Z. Feizabadi M. M. Babaei E. (2017). MgrB alterations mediate colistin resistance in *Klebsiella pneumoniae* isolates from Iran. *Front. Microbiol.* 8:2470. 10.3389/fmicb.2017.02470 29326662 PMC5741654

[B33] Haenni M. Poirel L. Kieffer N. Ch\^text{a}tre P. Saras E. Métayer V. (2016). Co-occurrence of extended spectrum β lactamase and *mcr-1* encoding genes on plasmids. *Lancet Infect. Dis.* 16 281–282. 10.1016/S1473-3099(16)00007-4 26774244

[B34] Hall T. A. (1999). BioEdit: a user-friendly biological sequence alignment editor and analysis program for Windows 95/98/NT. *Nucleic Acids Symp. Ser.* 41 95–98.

[B35] Hammerl J. A. Borowiak M. Schmoger S. Shamoun D. Grobbel M. Malorny B. (2018). *mcr-5* and a novel *mcr-5*.2 variant in *Escherichia coli* isolates from food and food-producing animals, Germany, 2010 to 2017. *J. Antimicrob. Chemother.* 73 1433–1435. 10.1093/jac/dky020 29444245

[B36] Hasman H. Hammerum A. M. Hansen F. Hendriksen R. S. Olesen B. Agersø Y. (2015). Detection of *mcr-1* encoding plasmid-mediated colistin-resistant *Escherichia coli* isolates from human bloodstream infection and imported chicken meat, Denmark 2015. *Euro Surveill.* 20:30085. 10.2807/1560-7917.ES.2015.20.49.30085 26676364

[B37] Hassen B. Abbassi M. S. Ruiz-Ripa L. Mama O. M. Hassen A. Torres C. (2020). High prevalence of *mcr-1* encoding colistin resistance and first identification of blaCTX-M-55 in ESBL/CMY-2-producing *Escherichia coli* isolated from chicken faeces and retail meat in Tunisia. *Int. J. Food Microbiol.* 318:108478. 10.1016/j.ijfoodmicro.2019.108478 31855787

[B38] Hembach N. Schmid F. Alexander J. Hiller C. Rogall E. T. Schwartz T. (2017). Occurrence of the *mcr-1* colistin resistance gene and other clinically relevant antibiotic resistance genes in microbial populations at different municipal wastewater treatment plants in Germany. *Front. Microbiol.* 8:1282. 10.3389/fmicb.2017.01282 28744270 PMC5504345

[B39] Inderbinen M. N. (2017). Assessment of the occurrence of MCR producing *Enterobacteriaceae* in Swiss and imported poultry meat. *J. Food Sci. Technol.* 1 137–141. 10.25177/JFST.1.4.5

[B40] Irrgang A. Roschanski N. Tenhagen B.-A. Grobbel M. Skladnikiewicz-Ziemer T. Thomas K. (2016). Prevalence of *mcr-1* in *E. coli* from livestock and food in Germany, 2010-2015. *PLoS One* 11:e0159863. 10.1371/journal.pone.0159863 27454527 PMC4959773

[B41] Izdebski R. Baraniak A. Bojarska K. Urbanowicz P. Fiett J. Pomorska-Wesołowska M. (2016). Mobile *mcr-1*-associated resistance to colistin in Poland. *J. Antimicrob. Chemother.* 71 2331–2333. 10.1093/jac/dkw261 27330064

[B42] Jacoby G. A. Strahilevitz J. Hooper D. C. (2014). Plasmid-mediated quinolone resistance. *Microbiol. Spectr.* 2 1–42. 10.1128/microbiolspec.PLAS-0006-2013 25584197 PMC4288778

[B43] Jayol A. Poirel L. Brink A. Villegas M.-V. Yilmaz M. Nordmann P. (2014). Resistance to colistin associated with a single amino acid change in protein PmrB among *Klebsiella pneumoniae* isolates of worldwide origin. *Antimicrob. Agents Chemother.* 58 4762–4766. 10.1128/AAC.00084-14 24914122 PMC4136042

[B44] Johnson T. J. Logue C. M. Johnson J. R. Kuskowski M. A. Sherwood J. S. Barnes H. J. (2012). Associations between multidrug resistance, plasmid content, and virulence potential among extraintestinal pathogenic and commensal *Escherichia coli* from humans and poultry. *Foodborne Pathog. Dis.* 9 37–46. 10.1089/fpd.2011.0961 21988401 PMC3250628

[B45] Jørgensen S. B. Søraas A. Arnesen L. S. Leegaard T. Sundsfjord A. Jenum P. A. (2017). First environmental sample containing plasmid-mediated colistin-resistant ESBL-producing *Escherichia coli* detected in Norway. *APMIS* 125 822–825. 10.1111/apm.12720 28640456

[B46] Kieffer N. Aires-de-Sousa M. Nordmann P. Poirel L. (2017). High rate of *mcr-1*-producing *Escherichia coli* and *Klebsiella pneumoniae* among pigs, Portugal. *Emerg. Infect. Dis.* 23 2023–2029. 10.3201/eid2312.170883 29148380 PMC5708242

[B47] Kluytmans-van, den Bergh M. F. Huizinga P. Bonten M. J. Bos M. Bruyne K. (2016). Presence of *mcr-1*-positive *Enterobacteriaceae* in retail chicken meat but not in humans in the Netherlands since 2009. *Euro Surveill.* 21:30149. 10.2807/1560-7917.ES.2016.21.9.30149 26967540

[B48] Kneis D. Berendonk T. U. Heß S. (2019). High prevalence of colistin resistance genes in German municipal wastewater. *Sci. Total Environ.* 694:133454. 10.1016/j.scitotenv.2019.07.260 31398645

[B49] Koyama Y. Kurosawa A. Tuchiya A. Takahisada K. (1950). A new antibiotic “colistin” produced by spore-forming soil bacteria. *J. Antibiot.* 3 457–458.

[B50] Leung L. M. Cooper V. S. Rasko D. A. Guo Q. Pacey M. P. McElheny C. L. (2017). Structural modification of LPS in colistin-resistant, KPC-producing *Klebsiella pneumoniae*. *J. Antimicrob. Chemother.* 72 3035–3042. 10.1093/jac/dkx234 28961916 PMC5890713

[B51] Li R. Xie M. Zhang J. Yang Z. Liu L. Liu X. (2017). Genetic characterization of *mcr-1*-bearing plasmids to depict molecular mechanisms underlying dissemination of the colistin resistance determinant. *J. Antimicrob. Chemother.* 72 393–401. 10.1093/jac/dkw411 28073961

[B52] Lin Y. Dong X. Wu J. Rao D. Zhang L. Faraj Y. (2020). Metadata analysis of *mcr-1*-bearing plasmids inspired by the sequencing evidence for horizontal transfer of antibiotic resistance genes between polluted river and wild birds. *Front. Microbiol.* 11:352. 10.3389/fmicb.2020.00352 32210943 PMC7076156

[B53] Liu Y.-Y. Wang Y. Walsh T. R. Yi L.-X. Zhang R. Spencer J. (2016). Emergence of plasmid-mediated colistin resistance mechanism *mcr-1* in animals and human beings in China: a microbiological and molecular biological study. *Lancet Infect. Dis.* 16 161–168. 10.1016/S1473-3099(15)00424-7 26603172

[B54] Magiorakos A.-P. Srinivasan A. Carey R. B. Carmeli Y. Falagas M. E. Giske C. G. (2012). Multidrug-resistant, extensively drug-resistant and pandrug-resistant bacteria: an international expert proposal for interim standard definitions for acquired resistance. *Clin. Microbiol. Infect.* 18 268–281. 10.1111/j.1469-0691.2011.03570.x 21793988

[B55] Malhotra-Kumar S. Xavier B. B. Das A. J. Lammens C. Hoang H. T. T. Pham N. T. (2016). Colistin-resistant *Escherichia coli* harbouring *mcr-1* isolated from food animals in Hanoi, Vietnam. *Lancet Infect. Dis.* 16 286–287. 10.1016/S1473-3099(16)00014-1 26774248

[B56] Moskowitz S. M. Brannon M. K. Dasgupta N. Pier M. Sgambati N. Miller A. K. (2012). PmrB mutations promote polymyxin resistance of *Pseudomonas aeruginosa* isolated from colistin-treated cystic fibrosis patients. *Antimicrob. Agents Chemother.* 56 1019–1030. 10.1128/AAC.05829-11 22106224 PMC3264203

[B57] Nation R. L. Li J. (2009). Colistin in the 21st century. *Curr. Opin. Infect. Dis.* 22 535–543. 10.1097/QCO.0b013e328332e672 19797945 PMC2869076

[B58] Nordmann P. Jayol A. Poirel L. (2016). A universal culture medium for screening polymyxin-resistant gram-negative isolates. *J. Clin. Microbiol.* 54 1395–1399. 10.1128/JCM.00446-16 26984971 PMC4844728

[B59] OIE (2018). *List of Antimicrobial Agents of Veterinary Importance.* Available online at: https://www.oie.int/fileadmin/Home/eng/Our_scientific_expertise/docs/pdf/AMR/A_OIE_List_antimicrobials_July2019.pdf (accessed September 19, 2019).

[B60] Olaitan A. O. Diene S. M. Kempf M. Berrazeg M. Bakour S. Gupta S. K. (2014). Worldwide emergence of colistin resistance in *Klebsiella pneumoniae* from healthy humans and patients in Lao PDR, Thailand, Israel, Nigeria and France owing to inactivation of the PhoP/PhoQ regulator mgrB: an epidemiological and molecular study. *Int. J. Antimicrob. Agents* 44 500–507. 10.1016/j.ijantimicag.2014.07.020 25264127

[B61] Poirel L. Kieffer N. Nordmann P. (2017). In vitro study of *ISApl1*-mediated mobilization of the colistin resistance gene *mcr-1*. *Antimicrob. Agents Chemother.* 61:e00127-17. 10.1128/AAC.00127-17 28416554 PMC5487687

[B62] Quesada A. Porrero M. C. Téllez S. Palomo G. García M. Domínguez L. (2015). Polymorphism of genes encoding PmrAB in colistin-resistant strains of *Escherichia coli* and *Salmonella enterica* isolated from poultry and swine. *J. Antimicrob. Chemother.* 70 71–74. 10.1093/jac/dku320 25150146

[B63] Rebelo A. R. Bortolaia V. Kjeldgaard J. S. Pedersen S. K. Leekitcharoenphon P. Hansen I. M. (2018). Multiplex PCR for detection of plasmid-mediated colistin resistance determinants, *mcr-1*, *mcr-2*, *mcr-3*, *mcr-4* and *mcr-5* for surveillance purposes. *Euro Surveill.* 23:17-00672. 10.2807/1560-7917.ES.2018.23.6.17-00672 29439754 PMC5824125

[B64] Roberts M. C. (2003). Tetracycline therapy: update. *Clin. Infect. Dis.* 36 462–467. 10.1086/367622 12567304

[B65] Savin M. Bierbaum G. Hammerl J. A. Heinemann C. Parcina M. Sib E. (2020a). Antibiotic-resistant bacteria and antimicrobial residues in wastewater and process water from German pig slaughterhouses and their receiving municipal wastewater treatment plants. *Sci. Total Environ.* 727:138788. 10.1016/j.scitotenv.2020.138788 32498197

[B66] Savin M. Bierbaum G. Hammerl J. A. Heinemann C. Parcina M. Sib E. (2020b). Isolation and characterization of ESKAPE-bacteria and ESBL-producing *E. coli* from waste- and process water of German poultry slaughterhouses. *Appl. Environ. Microbiol.* 86:e02748-19. 10.1128/AEM.02748-19 32033950 PMC7117925

[B67] Schrauwen E. J. A. Huizinga P. van Spreuwel N. Verhulst C. Kluytmans-van den Bergh M. F. Q. Kluytmans J. A. J. W. (2017). High prevalence of the *mcr-1* gene in retail chicken meat in the Netherlands in 2015. *Antimicrob. Resist. Infect. Control* 6:83. 10.1186/s13756-017-0242-8 28828173 PMC5563067

[B68] Snesrud E. He S. Chandler M. Dekker J. P. Hickman A. B. McGann P. (2016). A model for transposition of the colistin resistance gene *mcr-1* by IS*Apl1*. *Antimicrob. Agents Chemother.* 60 6973–6976. 10.1128/AAC.01457-16 27620479 PMC5075121

[B69] Sun J. Li X.-P. Fang L.-X. Sun R.-Y. He Y.-Z. Lin J. (2018a). Co-occurrence of *mcr-1* in the chromosome and on an IncHI2 plasmid: persistence of colistin resistance in *Escherichia coli*. *Int. J. Antimicrob. Agents* 51 842–847. 10.1016/j.ijantimicag.2018.01.007 29371103

[B70] Sun J. Zhang H. Liu Y.-H. Feng Y. (2018b). Towards understanding MCR-like colistin resistance. *Trends Microbiol.* 26 794–808. 10.1016/j.tim.2018.02.006 29525421

[B71] Veldman K. van Essen-Zandbergen A. Rapallini M. Wit B. Heymans R. van Pelt W. (2016). Location of colistin resistance gene *mcr-1* in *Enterobacteriaceae* from livestock and meat. *J. Antimicrob. Chemother.* 71 2340–2342. 10.1093/jac/dkw181 27246233

[B72] Villa L. García-Fernández A. Fortini D. Carattoli A. (2010). Replicon sequence typing of IncF plasmids carrying virulence and resistance determinants. *J. Antimicrob. Chemother.* 65 2518–2529. 10.1093/jac/dkq347 20935300

[B73] Wang C. Feng Y. Liu L. Wei L. Kang M. Zong Z. (2020). Identification of novel mobile colistin resistance gene *mcr-10*. *Emerg. Microbes Infect.* 9 508–516. 10.1080/22221751.2020.1732231 32116151 PMC7067168

[B74] Wang X. Wang Y. Zhou Y. Li J. Yin W. Wang S. (2018). Emergence of a novel mobile colistin resistance gene, *mcr-8*, in NDM-producing *Klebsiella pneumoniae*. *Emerg. Microbes Infect.* 7:122 doi: 10.1038/s41426-018-01 24-z 10.1038/s41426-018-0124-z PMC6030107 29970891

[B75] Webb H. E. Granier S. A. Marault M. Millemann Y. den Bakker H. C. Nightingale K. K. (2016). Dissemination of the *mcr-1* colistin resistance gene. *Lancet Infect. Dis.* 16 144–145. doi: 10.1016/S1473-3099(15)005 38-1 26711363 10.1016/S1473-3099(15)00538-1

[B76] World Health Organization (2019). *Critically Important Antimicrobials for Human Medicine: Ranking of Medically Important Antimicrobials for Risk Management of Antimicrobial Resistance due to Non-Human Use.* Geneva: World Health Organization.

[B77] Xavier B. B. Lammens C. Ruhal R. Kumar-Singh S. Butaye P. Goossens H. (2016). Identification of a novel plasmid-mediated colistin-resistance gene, *mcr-2*, in *Escherichia coli*, Belgium, June 2016. *Euro Surveill.* 21:30442. 10.2807/1560-7917.ES.2016.21.27.30280 27416987

[B78] Yang Y.-Q. Li Y.-X. Lei C.-W. Zhang A.-Y. Wang H.-N. (2018). Novel plasmid-mediated colistin resistance gene mcr-7.1 in *Klebsiella pneumoniae*. *J. Antimicrob. Chemother.* 73 1791–1795. 10.1093/jac/dky111 29912417

[B79] Yin W. Li H. Shen Y. Liu Z. Wang S. Shen Z. (2017). Novel plasmid-mediated colistin resistance gene *mcr-3* in *Escherichia coli*. *mBio* 8:e00543-17. 10.1128/mBio.00543-17 28655818 PMC5487729

[B80] Zhang J. Wang J. Chen L. Yassin A. K. Kelly P. Butaye P. (2018). Housefly (*Musca domestica*) and blow fly (*Protophormia terraenovae*) as vectors of bacteria carrying colistin resistance genes. *Appl. Environ. Microbiol.* 84:e01736-17. 10.1128/AEM.01736-17 29030447 PMC5734023

[B81] Zhang P. Bai L. Li Y. Wang Z. Li R. (2019). Loss of mcr genes mediated by plasmid elimination and IS*Apl1*. *Antimicrob. Agents Chemother.* 63:e01002-19. 10.1128/AAC.01002-19 31209010 PMC6709468

[B82] Zheng B. Dong H. Xu H. Lv J. Zhang J. Jiang X. (2016). Coexistence of *mcr-1* and NDM-1 in clinical *Escherichia coli* isolates. *Clin. Infect. Dis.* 63 1393–1395. 10.1093/cid/ciw553 27506685

[B83] Zheng B. Yu X. Xu H. Guo L. Zhang J. Huang C. (2017). Complete genome sequencing and genomic characterization of two *Escherichia coli* strains co-producing *mcr-1* and NDM-1 from bloodstream infection. *Sci. Rep.* 7:17885. 10.1038/s41598-017-18273-2 29263349 PMC5738369

[B84] Zhong Y.-M. Liu W.-E. Zheng Z.-F. (2019). Epidemiology and molecular characterization of *mcr-1* in *Escherichia coli* recovered from patients with bloodstream infections in Changsha, Central China. *Infect. Drug Resist.* 12 2069–2076. 10.2147/IDR.S209877 31372014 PMC6634265

[B85] Zhuge X. Ji Y. Tang F. Sun Y. Jiang M. Hu W. (2019). Population structure and antimicrobial resistance traits of avian-origin *mcr-1*-positive *Escherichia coli* in Eastern China, 2015 to 2017. *Transbound Emerg. Dis.* 66 1920–1929. 10.1111/tbed.13222 31059196

[B86] Zurfluh K. Tasara T. Poirel L. Nordmann P. Stephan R. (2016). Draft genome sequence of *Escherichia coli* S51, a chicken isolate harboring a chromosomally encoded *mcr-1* gene. *Genome Announc.* 4:e00796-16. 10.1128/genomeA.00796-16 27491979 PMC4974331

[B87] Zurfuh K. Poirel L. Nordmann P. Nüesch-Inderbinen M. Hächler H. Stephan R. (2016). Occurrence of the plasmid-borne *mcr-1* colistin resistance gene in extended-spectrum-β-lactamase-producing *Enterobacteriaceae* in river water and imported vegetable samples in Switzerland. *Antimicrob. Agents Chemother.* 60 2594–2595. 10.1128/AAC.00066-16 26883696 PMC4808203

